# Diversity and evolution of B-family DNA polymerases

**DOI:** 10.1093/nar/gkaa760

**Published:** 2020-09-25

**Authors:** Darius Kazlauskas, Mart Krupovic, Julien Guglielmini, Patrick Forterre, Česlovas Venclovas

**Affiliations:** Institute of Biotechnology, Life Sciences Center, Vilnius University, Saulėtekio av. 7, Vilnius 10257, Lithuania; Archaeal Virology Unit, Department of Microbiology, Institut Pasteur, 25 rue du Docteur Roux, Paris 75015, France; Hub de Bioinformatique et Biostatistique – Département Biologie Computationnelle, Institut Pasteur, USR 3756 CNRS, Paris, France; Archaeal Virology Unit, Department of Microbiology, Institut Pasteur, 25 rue du Docteur Roux, Paris 75015, France; Institute of Biotechnology, Life Sciences Center, Vilnius University, Saulėtekio av. 7, Vilnius 10257, Lithuania

## Abstract

B-family DNA polymerases (PolBs) represent the most common replicases. PolB enzymes that require RNA (or DNA) primed templates for DNA synthesis are found in all domains of life and many DNA viruses. Despite extensive research on PolBs, their origins and evolution remain enigmatic. Massive accumulation of new genomic and metagenomic data from diverse habitats as well as availability of new structural information prompted us to conduct a comprehensive analysis of the PolB sequences, structures, domain organizations, taxonomic distribution and co-occurrence in genomes. Based on phylogenetic analysis, we identified a new, widespread group of bacterial PolBs that are more closely related to the catalytically active N-terminal half of the eukaryotic PolEpsilon (PolEpsilonN) than to *Escherichia coli* Pol II. In Archaea, we characterized six new groups of PolBs. Two of them show close relationships with eukaryotic PolBs, the first one with PolEpsilonN, and the second one with PolAlpha, PolDelta and PolZeta. In addition, structure comparisons suggested common origin of the catalytically inactive C-terminal half of PolEpsilon (PolEpsilonC) and PolAlpha. Finally, in certain archaeal PolBs we discovered C-terminal Zn-binding domains closely related to those of PolAlpha and PolEpsilonC. Collectively, the obtained results allowed us to propose a scenario for the evolution of eukaryotic PolBs.

## INTRODUCTION

Cellular replicative DNA polymerases (Pols) are classified into three families of non-homologous enzymes, PolB, PolC and PolD, that synthesize DNA at the replication forks of eukaryotes, bacteria and most archaea, respectively (1,2). Among replicative polymerases, PolBs are the most widespread, found in all domains of life and several lineages of DNA viruses (3). PolBs can be subdivided into three major apparently monophyletic assemblages based on the primer they use. Protein-primed PolBs (pPolBs) replicate small linear genomes of viruses and selfish mobile genetic elements (4–6). The PolBs that require a pre-existing nucleic acid (RNA or DNA) primer participate in genome replication and repair in all living organisms and their viruses (3). The third assemblage includes the recently identified group of primer-independent PolBs (piPolBs) encoded by bacterial and mitochondrial mobile genetic elements and capable of template-dependent *de novo* DNA synthesis (7).

**Figure 1.**
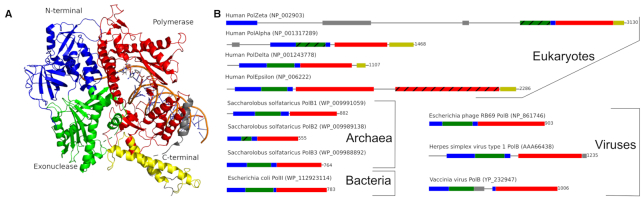
Structure and domain architecture of PolBs. (**A**) Ternary complex of human PolDelta catalytic subunit with an incoming nucleotide (PDB: 6tny). (**B**) Domain architecture of different PolBs. N-terminal, exonuclease, polymerase, C-terminal and other domains are shown in dark blue, dark green, red, yellow and gray, respectively.

PolBs have three common domains, namely, N-terminal, Exonuclease (Exo) and DNA polymerase (Pol) (Figure 1). The role of the N-terminal domain is not completely understood. It was found to be important for uracil recognition in archaeal, but not in eukaryotic enzymes (8). The Exo domain uses its 3′–5′ exonuclease activity for proofreading, whereas the right hand-shaped Pol domain performs the actual template-dependent DNA synthesis (9). The PolBs have six conserved regions (I–VI): three sequence motifs in Exo (ExoI, ExoII, ExoIII) domain and three in Pol (MotifA, MotifB, MotifC) domain (10) (Supplementary Figure S1). PolBs have been divided into many monophyletic subfamilies largely confined to a specific cellular domain. Eukaryotes have four multimeric PolBs, namely, Alpha (PolAlpha), Delta (PolDelta), Zeta (PolZeta) and Epsilon (PolEpsilon). Each eukaryotic PolB comprises a distinct catalytic PolB subunit (also referred to as A-subunit), a regulatory subunit (B-subunit), and an assortment of accessory subunits (2,11). The four Pols have different functions in the cell. PolAlpha participates in the initiation of DNA synthesis, whereas PolEpsilon and PolDelta are responsible for the bulk synthesis of leading and lagging DNA strands, respectively (12). PolZeta is a low fidelity enzyme involved in translesion DNA synthesis primarily by extending the strand past the lesion (13). Catalytic subunits of both PolAlpha and PolZeta have an inactivated Exo domain (Figure 1B). The A-subunit of PolEpsilon stands out among other eukaryotic PolBs. First, it represents a fusion of two distinct PolBs, one corresponding to the catalytically active N-terminal module (PolEpsilonN) and the other one to the inactivated C-terminal module (PolEpsilonC) (14). Interestingly, in yeast, PolEpsilonC, which interacts with the replicative CMG (Cdc45–MCM–GINS) helicase, is essential, whereas PolEpsilonN is not (15,16). Second, PolEpsilonN features a novel P-domain, inserted into the palm subdomain and contributing to the high processivity of the polymerase (17). In addition, at the base of the P-domain, PolEpsilonN has the iron–sulfur (Fe–S) cluster-binding cysteine motif (CysX), important for polymerase function (18). Both P-domain and the CysX motif are present in PolEpsilon orthologs, but not in other PolBs. Notably, the catalytic subunits of all four eukaryotic PolBs have C-terminal domains (CTDs) (Figure 1). CTDs mediate binding to the corresponding B-subunits and each harbors a pair of C4-type metal-binding motifs (CysA and CysB). Structural studies revealed that CTDs represent α-helical bundles with CysA and CysB motifs at the opposite ends. Previously, CysA and CysB motifs in all four catalytic subunits were thought to bind zinc ions (14). However, current biochemical and structural data indicates that only CysA motif binds Zn^2+^ in all four PolBs. The CysB motif in PolAlpha and PolEpsilon also binds Zn^2+^, but in PolDelta and PolZeta CysB binds the Fe–S cluster (11). Not surprisingly, CTD structures of PolAlpha and PolEpsilon are considerably more similar to each other than to the corresponding CTDs of PolDelta and PolZeta (19–23). So far, no metal-binding domains have been detected in archaeal PolBs (24).

The best characterized archaeal PolBs belong to the three groups, B1, B2 and B3, all of which are represented in *Saccharolobus solfataricus* (25) and are widespread in other archaea (24,26). By contrast, in Bacteria, only one group of PolBs is known; it is represented by *Escherichia coli* PolII participating in translesion synthesis (27). PolBs are also encoded by many DNA viruses with larger genomes, mainly belonging to the order *Caudovirales* (head-tailed viruses of Archaea and Bacteria), Nucleo-Cytoplasmic Large DNA Viruses (NCLDV) and several other families of eukaryotic viruses such as baculoviruses and herpesviruses. In a recent study, Mushegian and colleagues showed that PolBs encoded by eukaryotic viruses form two clades, one including NCLDVs (excluding *Poxviridae* and *Asfarviridae*), *Hytrosaviridae* and *Herpesviridae* and the other one containing *Alloherpesviridae*, *Malacoherpesviridae*, *Poxviridae*, *Baculoviridae* and *Nimaviridae* (28).

Phylogenetic analyses of cellular PolBs and their viral homologs (3,24) suggested different origins of the PolEpsilonN from the rest of eukaryotic PolBs. However, deep branches in all previous studies were not well resolved. Here, to unravel the evolutionary history of PolBs, we collected a representative set of these proteins from archaea, bacteria, eukaryotes and viruses, and performed a comprehensive analysis of their sequences, structures, domain organizations, taxonomic distribution and co-occurrence in genomes. As a result, we defined and characterized six new groups of archaeal PolBs and a new group of bacterial PolBs, which appears to be related to the catalytically active N-terminal module of the eukaryotic PolEpsilon. We also uncovered the similarity of the catalytically inactive PolEpsilon C-terminal module to PolAlpha. Finally, we discovered that two novel groups of archaeal PolBs have C-terminal metal-binding domains, closely related to those present in eukaryotic PolAlpha and PolEpsilon. Collectively, the results of this study allowed us to propose a scenario for the evolution of eukaryotic PolBs.

## MATERIALS AND METHODS

### Databases

For sequence searches we used non-redundant (NR) databases: NCBI’s NR, UniProt, metagenomic databases (KEGG MGENES, Uncultivated Bacteria and Archaea (UBA) metagenomes (29), Integrated Microbial Genomes (IMG) (30), MGnify (31)) and sequences from Magroviruses (32). For sensitive profile-profile searches PDB (33) and Pfam 32.0 (34) databases were used.

### Structure similarity searches

To analyze structural similarity between PolBs, we performed searches using Dali server (35) and the structure of yeast DNA polymerase Delta (PDB: 3iay, chain A) as a query. Hits to the PDB database filtered to 90% identity, having >400 locally aligned residues, were forwarded to Dali pairwise comparison server. Structure similarity dendrogram was used to visualize the results.

### Sequence searches and clustering

A set of queries for the initial sequence search were collected as follows. First, the structure of eukaryotic PolDelta (pdb: 3iay, chain A) was used as a query for Dali search against pdb90 DB. Next, polymerase domains were extracted using a structure-based multiple sequence alignment and in turn were used as queries for the three-iteration Jackhmmer (36) searches against the UniRef50 database. Hits with E-value lower than 1e–03 were extracted and clustered with CLANS (37). After removal of false positives (sequences that did not have the polymerase domain) and singletons (sequences that did not have connections to other sequences at CLANS *P*-value of 1e–08) we were left with 4428 sequences. In order to use the most accurate multiple alignment modes in MAFFT (38) we had to decrease our initial dataset. Thus, we selected only the representative members of sub-clusters which had >200 sequences. To collect homologs for DP1 phylogenetic analysis, six iterations of Jackhmmer using human PolDelta B-subunit (AAC50216) as a query were run against UniRef50. Hits with *E*-value lower than 1e–03 were extracted and clustered with CLANS at 1e–10, highly divergent sequences discarded, groups identified and sequences to be aligned with MAFFT were extracted. To cluster CTDs, CLANS with the PSI-BLAST option was used. For profile construction, two PSI-BLAST iterations with the 1e–03 inclusion threshold were run against the NCBI env_nr database, supplemented with the sequences to be compared. CTDs were extracted from sequences of PolBs which were found during the initial search against the UniRef50 database. DP2 CTDs were recovered after three iterations of Jackhmmer against UniRef50 and archaeal genomes databases using DP2 from *Pyrococcus abyssi* as a query. Searches for small groups (<25 members) were performed against NCBI’s NR and MGnify databases.

### Multiple sequence alignments

Multiple sequence alignments for phylogenetic analysis were constructed using MAFFT. Specifically, alignment for the trees shown in Figure 3, Supplementary Figure S8 and Figure 7 were generated using MAFFT with options ‘- -ep 0.123 - -localpair’. To better align divergent sequences (Supplementary Figure S9), MAFFT with structural alignments (MAFFT-DASH) and additional homology searches (mafft-homologs.rb) were used (command line: mafft-homologs.rb -l -d uniref50.fasta -o ‘- -thread 8 - -threadtb 5 - -threadit 0 - -reorder - -dash - -maxiterate 1000 - -retree 1 - -localpair - -ep 0.123’ -a 50 -e 1.0e-20). The latter strategy was shown to improve the quality of the alignments (39). However, this strategy uses DASH server, which limits alignment size to 750 sequences. Thus, our initial 2813 sequence set had to be shrunk to a set of 639 sequences by randomly selecting every sixth sequence from groups larger than 60 members.

### Phylogenetic analysis

Tree building for Figure 3 was done iteratively. Clades of an initial tree having long branches and(or) low branch support values were subjected to additional sequence searches in NR and metagenomic databases. Next, newly found sequences were added to the dataset and both the alignment and the tree were rebuilt. If clades did not improve they were deleted from the alignment. The final set contained 2813 sequences (Supplementary Table S5). TrimAl with parameters ‘-gt 0.1’ was used to trim alignments and IQtree (40) (parameters: ‘-alrt 1000 -bb 1000’; automatic model selection) to build the trees.

### Taxonomic distribution

To determine taxonomic spread of PolBs, sequence profile searches were performed against prokaryotic, eukaryotic and viral proteomes (https://data.ace.uq.edu.au/public/misc_downloads/annotree/r89/, ftp://ftp.ensemblgenomes.org/pub/release-39/ and ftp://ftp.ncbi.nlm.nih.gov/refseq/release/viral/, respectively). Sequence profiles were built with HMMER from multiple sequence alignments of all PolB groups determined in the phylogenetic analysis step. To visualize the results, trees of prokaryotic phyla from AnnoTree (41) and iTol web server (42) were used.

### Analysis of domain architectures

Boundaries of the DNA polymerase domain were determined from MAFFT multiple sequence alignments of PolBs. Next, sequence regions located both N and C terminally from the identified DNA polymerase domain were extracted and subjected to profile searches using HHsuite (43) or HHpred server (44) against PDB and Pfam 32.0 databases.

## RESULTS

### Sequence searches and clustering of PolB DNA polymerases

**Figure 2.**
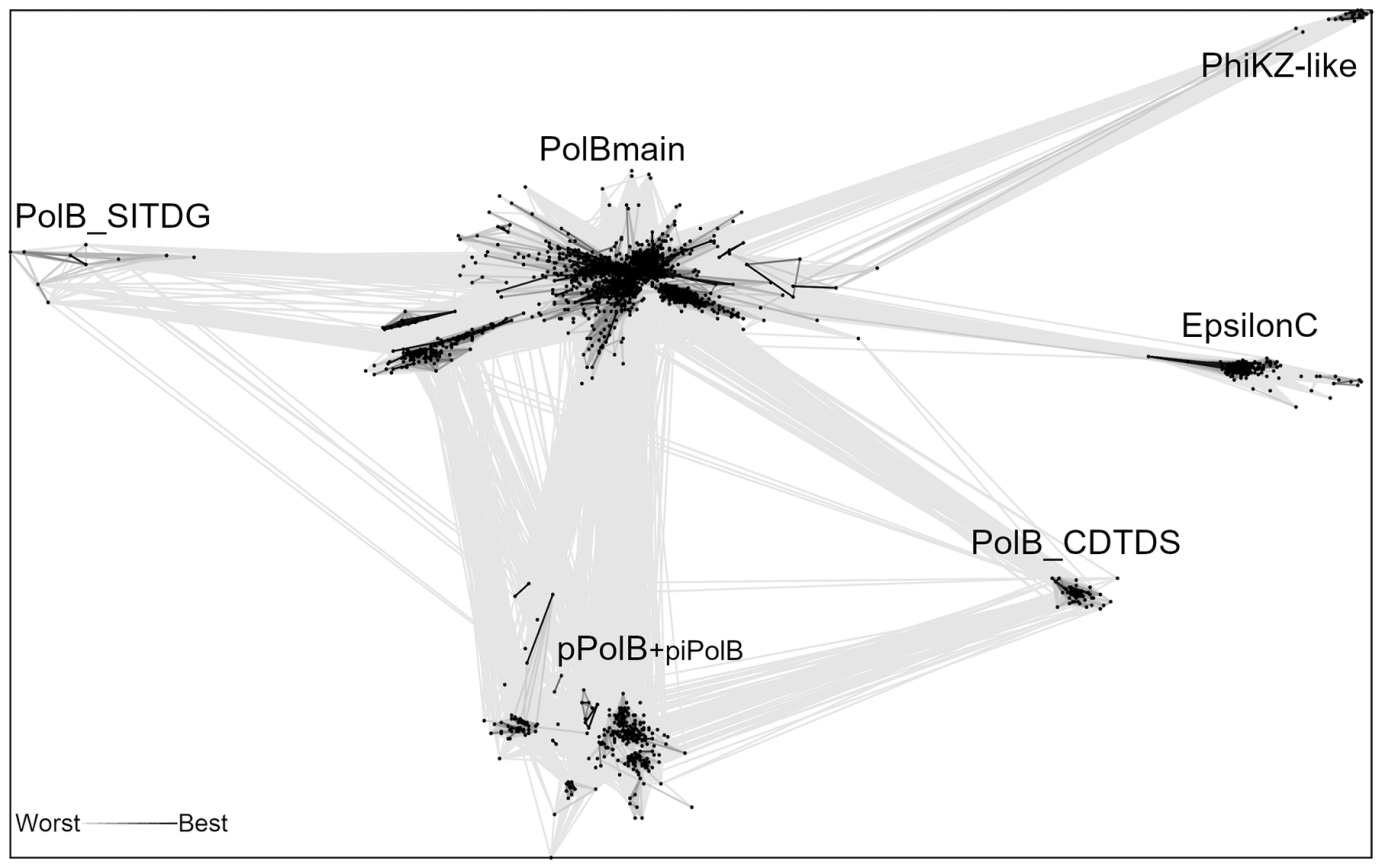
All-to-all comparison of B-family DNA polymerases with CLANS. Lines connect sequences with *P*-value ≤ 1e−05. Six resulting clusters are labeled. A smaller label for piPolB indicates that this group is represented by only three sequences.

To collect PolB homologs, we used sensitive sequence searches against UniRef50 queried with polymerase domains of PolBs for which high-resolution structures are available (Supplementary Table S1, Supplementary Figure S2). Full-length sequences of the resulting matches (4438 in total) were extracted and PolEpsilon sequences were split into two parts (PolEpsilonN and PolEpsilonC). Next, all these sequences were clustered with CLANS. After removal of sequence fragments shorter than 200 residues, false positives (i.e. sequences that did not have the PolB domain) and singletons (sequences that did not have connections at CLANS *P*-values of 1e–05), the final dataset included 3144 PolB sequences (Supplementary Table S2). Analysis of the resulting networks led to the identification of six clusters (Figure 2). The two largest ones contained 2784 and 280 sequences, respectively. The latter (thereafter called the pPolB+piPolB cluster) included all protein-primed DNA polymerases from casposons and viruses (e.g. Enterobacteria phage PRD1, bacteriophage GA-1, Acidianus bottle-shaped virus) and piPolBs. All catalytically active experimentally characterized PolBs belong to the largest cluster, thereafter called PolBmain group. Among the remaining four clusters of divergent PolBs, three (PhiKZ, PolEpsilonC and PolB_SITDG) showed distant relationship to the PolBmain group, whereas the PolB_CDTDS cluster contains sequences related to both PolBmain and pPolB groups (Figure 2). We have previously shown that PhiKZ phages encode a divergent homolog of the phage T4 DNA polymerase (3). PolB_SITDG and PolB_CDTDS groups were named after conserved residues in their active site MotifC. In PolB_SITDG proteins, found in cyanobacteria and algae (Supplementary Table S2), the first conserved aspartate is substituted with a hydrophobic residue. A member of PolB_CDTDS group from *Aciduliprofundum boonei* T469 (Supplementary Table S2) was shown to be encoded by a casposon (45), a recently discovered group self-synthesizing mobile genetic elements integrated in bacterial and archaeal genomes (5). Structure and sequence similarity searches indicated that PolEpsilonC is most closely related to PolAlpha (Supplementary Table S3, Supplementary Figures S3 and S4). However, due to the high sequence divergence between the PolBs from different clusters, we decided to perform phylogenetic analyses only of the main PolB cluster (Figure 2).

### Phylogenetic analysis, taxonomic distribution and conserved features of PolBs

**Figure 3.**
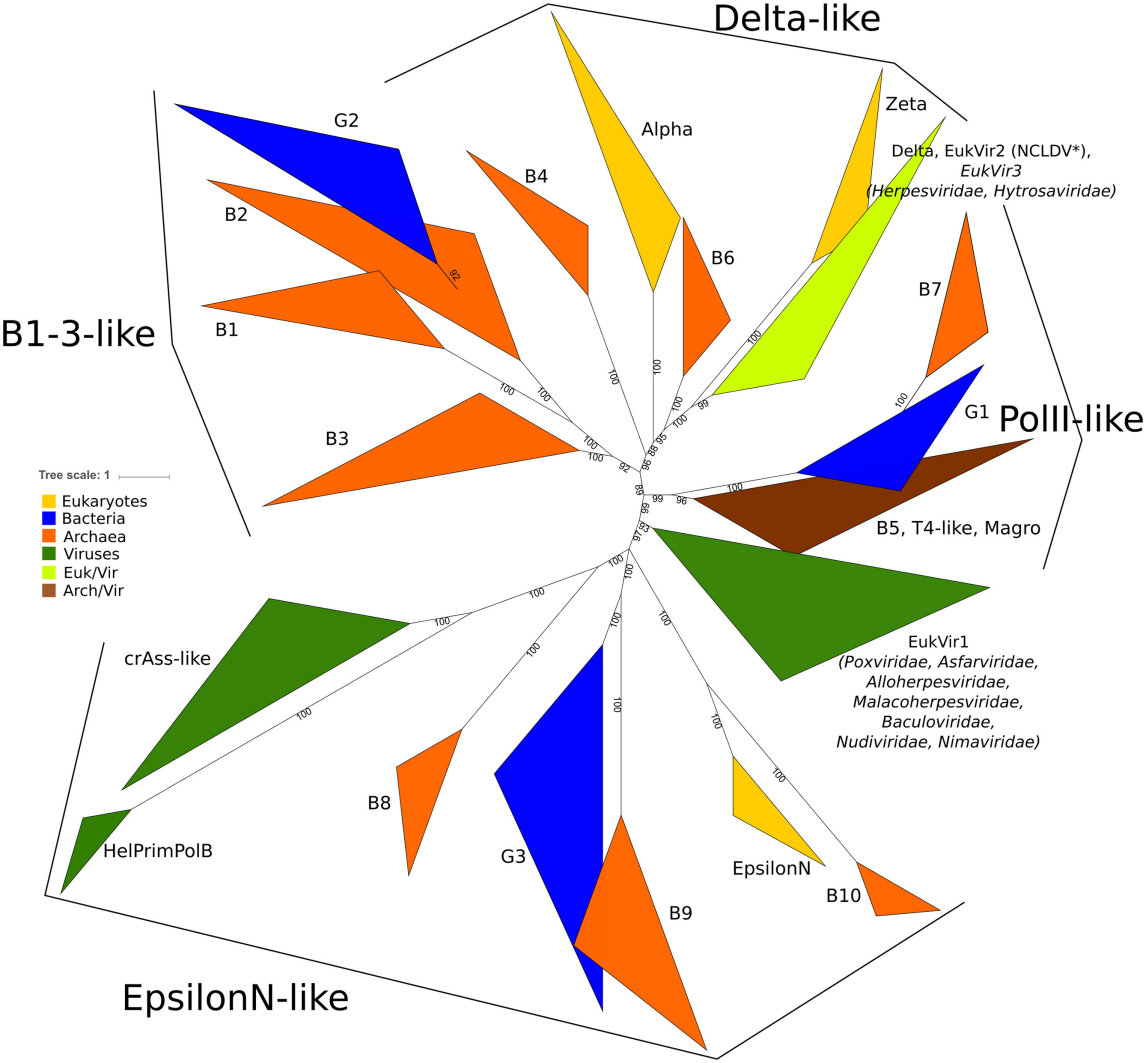
Phylogenetic tree of PolBs based on an accuracy-oriented MAFFT alignment. Asterisk marks a group of sequences from NCLDV except *Poxviridae* and *Asfarviridae*.

**Figure 4.**
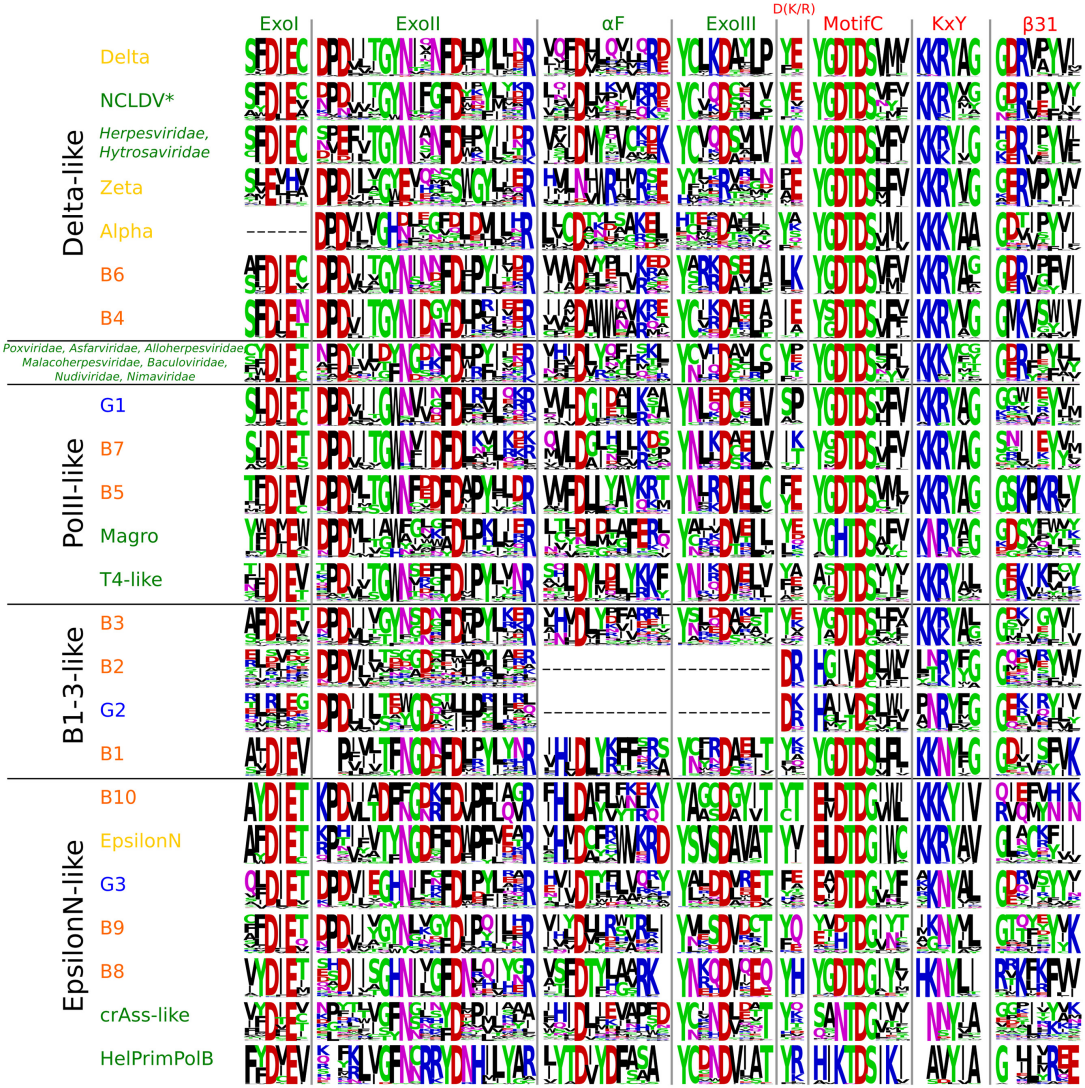
Conserved motifs of PolBs. Motifs were made using WebLogo from the alignment used for constructing phylogenetic tree shown in Figure 3. Group names are color-coded the same as in Figure 3. Names of motifs/regions are colored by domain (exonuclease, green; polymerase, red) and are either taken from the literature (7,69) or are named after the secondary structure elements of PolDelta (Supplementary Figure S1).

**Figure 5.**
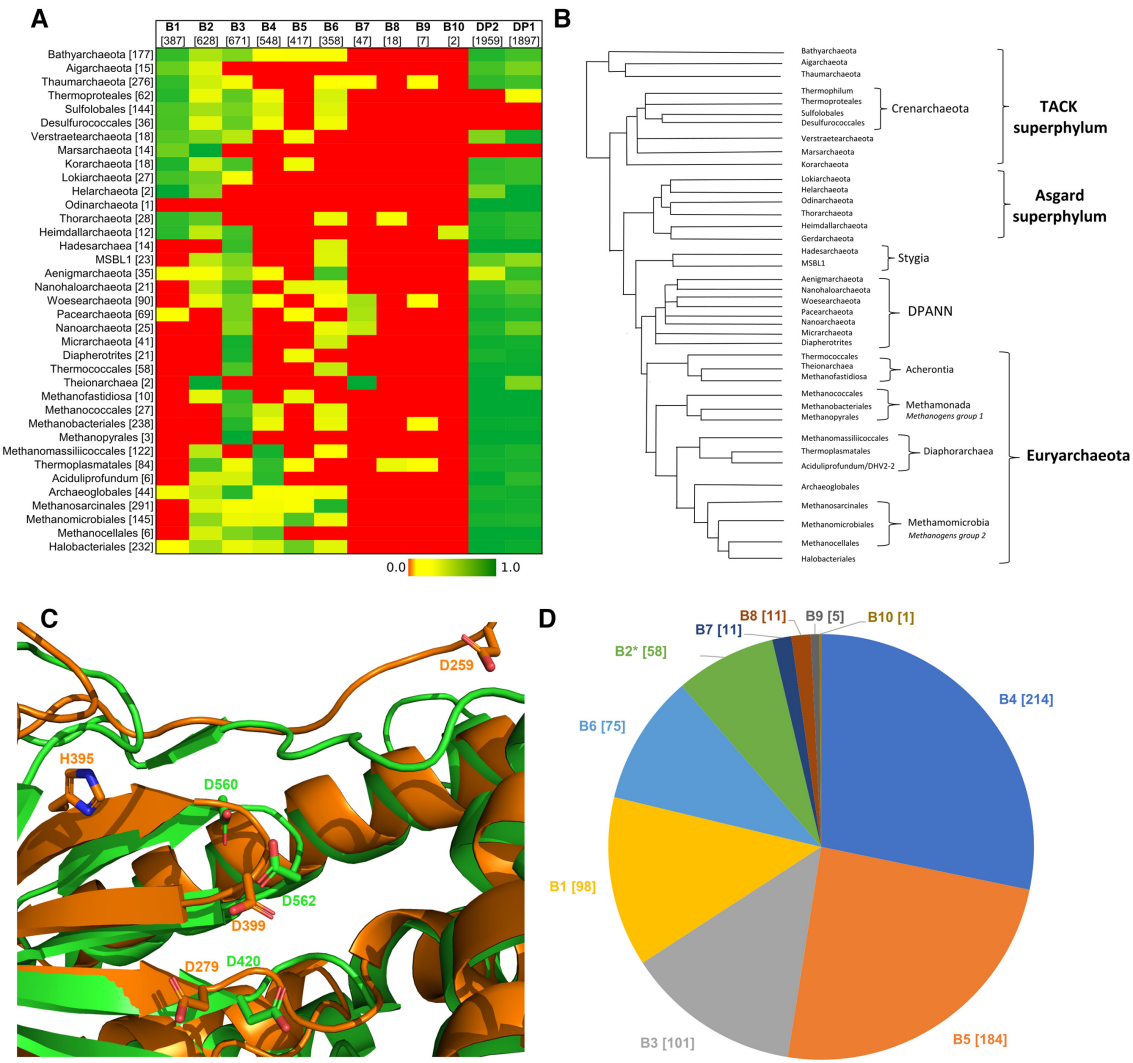
Archaeal PolBs. (**A**) Distribution of PolBs, PolD large (DP2) and small (DP1) subunits in archaeal genomes taken from GenBank (Supplementary Table S4). Numbers of species in a taxon and groups are shown in brackets. (**B**) The unrooted evolutionary tree of Archaea which is based on the schematic tree (70) updated according to recent phylogenetic analyses (49,71,72). (**C**) Model of B2 PolB (AAK41686) from *Saccharolobus solfataricus* P2 active site (orange) aligned to PolB3 from *Pyrobaculum calidifontis* (green color, PDB:5mdn). (**D**) B2 group members are often found together with other archaeal PolBs in the same organism. Co-occurrences are represented as a pie chart. Total of 628 genomes encode B2, 58 of them have B2 as a sole PolB (marked with an asterisk).

Although there were multiple recent attempts to build phylogenies of PolBs (3,24,28), our current work differs from the previous analyses in two major ways. First, our dataset was enriched by metagenomic sequences and contained nearly 3000 sequences (see Materials and Methods). Second, along with traditional multiple sequence alignment algorithms, we used a highly sensitive sequence alignment method which takes into account structural information (see Materials and Methods). The resulting tree for the main PolB cluster has five well-supported (IQtree UFB scores 92 and better) major clades, namely, B1–3-like, PolII-like, Delta-like, PolEpsilonN-like and EukVir1 (Figure 3). These clades are additionally strengthened by the comparison of conserved motifs (Figure 4). Each major clade includes several smaller subclades. Phylogenetic analysis indicates that the archaeal and viral sequences are the most diverse (present in four out of five clades), whereas bacterial and eukaryotic PolBs display more modest distribution (found in three and two clades, respectively). To analyze the distribution of PolB groups in prokaryotes, we constructed profile HMMs for each of the groups and searched the prokaryotic and viral genomes available in GenBank, RefSeq and the Genome Taxonomy Database (GTDB) (46). In addition to the well know B1–3, we defined seven groups of PolBs encoded in archaeal genomes and/or metagenome-assembled genomes (MAGs) and named them B4 (the largest) through B10 (the smallest) (Figure 5A and Supplementary Figure S5).

#### B1-3-like clade

The three major groups of archaeal PolBs, B1–3, form monophyletic subclades within a single major clade, B1–3-like (Figure 3). Consistent with the previous results (24), the B3 group is found in 30 out of 37 archaeal lineages and is the largest and most taxonomically diverse of all archaeal PolBs (Figure 5A). B1 and B3 are closely similar in regions β4, αF and MotifA (Supplementary Figure S6), whereas B1 and B2 display similarity in MotifA, MotifB and αW. A fourth subclade (G2), comprising bacterial PolBs, can be defined, but it is nested deeply within the archaeal B2 group (Figure 3). Notably, in B2 and G2 groups, ExoI is not conserved and ExoIII is missing altogether, implying that G2 has been horizontally transferred to Bacteria from Archaea, consistent with previous suggestion (24). Due to substitutions in the Exo and Pol active sites (Figure 4, MotifC has only one conserved Asp), B2 was considered to represent an inactivated group of archaeal PolBs (47). Indeed, most members of B-family have two aspartates (Supplementary Figure S1). Notably, however, although primer-independent PolBs also lack the first Asp residue in MotifC, they display highly efficient DNA polymerization activity (7). Furthermore, analysis of the multiple sequence alignment of B2/G2 PolBs revealed the presence of a highly conserved motif, D(K/R), specific to this group of PolBs (Figure 4). We hypothesized that the conserved aspartate in the D(K/R) motif might substitute for the first Asp in the MotifC. To test this hypothesis, we modeled the structure of experimentally characterized B2 member (AAK41686) from *S. solfataricus* P2 (25). It turned out that Asp from the D(K/R) motif is located in a flexible loop near the polymerase active site and thus could indeed replace the ‘missing’ Asp from MotifC (Figure 5C). Moreover, the histidine from MotifC is highly conserved in B2 and G2 groups (Figure 4) suggesting its possible role in the catalysis. Overall, such rearrangements of an active site may be an adaptation for carrying out a specialized function, namely, translesion synthesis (TLS). Indeed, it was shown that although B2 of *S. solfataricus* has only a weak DNA polymerase activity, it is able to bypass hypoxanthine, 8-oxoguanine and uracil lesions (25). Furthermore, it has been recently demonstrated that B2 of *S. islandicus* is the main DNA polymerase responsible for DNA damage tolerance and functions as a damage-inducible TLS enzyme solely responsible for targeted mutagenesis, facilitating GC to AT/TA conversions (48). Thus, despite substitutions in the active site, it appears that B2 and G2 PolB groups comprise active DNA polymerases involved in TLS. The repair function of B2 is further supported by its distribution in archaeal genomes. B2 is the only archaeal PolB often found with other groups (e.g. B5, B4, B1, B3) of PolBs (Figure 5D). Moreover, B2 is not found in Thermococcales (Figure 5A) that always have a member of B3, which in *Thermococcus kodakarensis* was found to be important for DNA repair and not for DNA replication (49). By contrast, B3 from euryarchaeon *Pyrococcus abyssi* and crenarchaeon *S. solfataricus* were shown to be involved in genome replication (50,51), although the main DNA polymerase responsible for the synthesis of the leading strand in *S. solfataricus* is B1 (50). Collectively, the available experimental data and the distribution of B1–3 groups in archaeal genomes suggest that B3 polymerases can have either DNA replication or DNA repair functions.

#### PolII-like clade

This clade is named after the well-known B-family member from *E. coli*. PolII-like clade has a unique ExoII motif (Figure 4, conserved residues ‘GWN’) containing a highly conserved tryptophan residue which corresponds to W216 in well-characterized PolB of phage RB69 and is located in the exonuclease active site, although its specific function is not known (52). *E. coli* PolII belongs to the largest group (G1) of the PolII-like clade. G1 members are mainly found in Proteobacteria (1964 species, 26% of all Proteobacteria (Supplementary Figure S7)). In our phylogenetic analysis, archaeal B7 group is nested within the bacterial G1 group and is found in several taxa phylogenetically related to Nanoarchaeota, namely, Woesearchaeota and Pacearchaeota which are part of the DPANN superphylum (Figure 5A and B). This suggests that B7 PolBs were acquired from bacteria and inherited vertically within the DPANN archaea.

Another large subclade within the PolII-like clade includes three groups, namely, B5, T4-like and Magro, all of which contain viral sequences (Supplementary Table S5). The B5 group is dominated by bona fide cellular sequences, with viral sequences being in minority (Supplementary Table S6; Haloviruses HF1, HF2, HCTV-1, HGTV-1, HVTV-1 and HRTV-5). B5 group is the fourth largest group of PolBs in Archaea (Figure 5A) and most often found in Halobacteriales and Methanomicrobiales (Supplementary Table S6). In *Halobacterium* sp. NRC-1, the B5 PolB was shown to be essential for cell viability along with PolD (27). The viral sequences in B5 display similarity to PolBs of Magroviruses, a group of metagenomically sequenced viruses associated with Marine Group II Euryarchaeota (32). T4-like group largely comprises PolBs of T4-like bacteriophages. Notably, DNA polymerases from T4 and RB69 phages are the most studied DNA polymerases in the B-family (171 out of 255 solved structures belong to T4-like phages; Supplementary Table S1). B5 and T4-like groups share similarities in MotifA (Supplementary Figure S6) and ExoIII (Figure 4). Interestingly, the replication apparatus of T4-like phages, namely, DNA polymerase, sliding clamp and clamp loader are structurally similar to those found in archaea and eukaryotes (53). Haloviruses and Magroviruses also encode these additional components of the replisome (3,53). These findings coupled with the observed limited distribution of B5 group in Archaea (Figure 5A), suggest that this group of PolBs may have evolved in viruses and was subsequently transferred to halophilic and methanogenic archaea.

#### EpsilonN-like clade

The most diverse supergroup of PolBs is the EpsilonN-like clade. It contains sequences from all domains of cellular organisms and viruses (Figure 3). Phylogenetic and motif analyses suggest that the N-terminal catalytically active domain of PolEpsilon is related to archaeal PolBs of group B10 and bacterial group G3. Most obvious synapomorphy is in MotifC where PolEpsiloN, B10 and G3 polymerases have a conserved glutamate residue (Figure 4). Less pronounced similarities are also found in motifs αF and ExoIII. G3 is the second largest PolB group in bacteria, which we describe here for the first time. It is also the most taxonomically diverse group of PolBs in bacteria, found in eight phyla, versus four phyla in the case of either G1 or G2 (Supplementary Figure S7). G3 is almost exclusively found in bacteria. The only two exceptions include G3 PolBs encoded by *Salinibacter* (54) and *Nostoc* phages (Supplementary Table S6). In both cases, the closest BLAST hits to the phage PolBs are from bacteria, suggesting that these G3 PolBs were acquired by phages from the respective hosts. By contrast, the origin of PolB of the B10 group is less clear since this PolB is only present in two genomes of Heimdallarchaea (LC3, B3) (Supplementary Table S5). B9 group, the second archaeal group in this clade, is a sister group to G3 (Figure 3). Most of the members of B9 (96%) are found in metagenomic databases with only two sequences being annotated (PSG96791 from Thermoplasmatales archaeon SW_10_69_26 and RLE38063 from Candidatus Woesearchaeota archaeon) (Supplementary Table S5). The third archaeal group in EpsilonN-like clade is B8. Unlike in the case of B9, an archaeal source of PolB of the B8 group is very likely, since these polymerases are present in all MAGs of Deep‐sea Hydrothermal Vent Euryarchaeota Group 1 (DHVEG‐1), making the contamination unlikely. However, the absence of this protein in *Thermoplasma acidophilum* is conspicuous. Groups crAss-like and HelPrimPolB are divergent members of the EpsilonN-like clade. The differences are profound in MotifC and KxY motifs (Figure 4). Members of the HelPrimPolB subgroup were previously shown to represent multidomain enzymes in which the polymerase domain is fused to the superfamily 3 helicase and PrimPol domains at their N-termini (3). Thus, this group was named based on its domain organization. Groups HelPrimPolB, crAss-like and B9 are prevalent in metagenomic datasets (Supplementary Table S5). Actually, the best-known member of the crAss-like group, crAssphage, was metagenomically sequenced from the human gut samples (55), whereas related phages were discovered in diverse environmental samples (56).

#### Delta-like and EukVir1 clades

Three out of four eukaryotic PolBs belong to the Delta-like clade (Figure 3). Proteins in this clade share four characteristic regions. Three of these motifs, namely, ExoI, ExoIII and MotifA contain conserved cysteine residues (Supplementary Figure S8B). Despite the presence of a unique subdomain upstream of the inactivated exonuclease domain in PolZeta (Figure 1), PolDelta and PolZeta have closely similar common regions (Supplementary Figure S6) and form a single branch in the phylogenetic trees (Figure 3 and Supplementary Figure S9). PolDelta is also closely related to DNA polymerases of eukaryotic viruses (Figure 3, EukVir(2/3)). EukVir2 contains viruses of the NCLDV assemblage (except *Poxviridae* and *Asfarviridae*) and EukVir3 includes members of the families *Herpesviridae* and *Hytrosaviridae*, consistent with the previous results (57). Delta-like clade contains two archaeal PolB groups, B4 and B6, found in Thermoplasmatota and Aenigmarchaeota/Methanosarcina, respectively (Figure 5A). The PolBs corresponding to the latter groups are also encoded by genomes obtained from cultivated organisms (*Thermoplasma acidophilum* and group 2 methanogens, respectively), confirming the archaeal source of the corresponding sequences. While the B4 group is positioned at the root of the Delta-like clade in both trees (Figure 3 and Supplementary Figure S9), the position of the B6 group varies. It is known that resolution and quality of the clades can be improved by building alignments with a larger number of informative sites for a subset of taxa or a bigger tree itself can be made from the combined smaller trees (58). Thus, to get a better understanding of the relationships between different groups in the Delta-like clade, we built a separate phylogeny. In addition to sequences from the Delta-like clade, we added EukVir1 sequences because this group contains Vaccinia PolE9, which is structurally closer to PolDelta and PolAlpha than to archaeal PolBs (59). B3 sequences were considered as an outgroup. In the resulting tree, EukVir1 forms a sister group to EukVir2–3, PolDelta and PolZeta (Supplementary Figure S8A). EukVir1 also shares similar motifs with the latter groups (Supplementary Figure S8B), suggesting that its position as a sister group to the EpsiloN-like clade in larger trees (Figure 3 and Supplementary Figure S9) is a technical artifact due to high divergence of EukVir1 and fewer informative sites in a larger multiple sequence alignment. Polymerases of the Delta-like clade still branch as a sister group to those of the B6 (Supplementary Figure S8A), suggesting that eukaryotic PolAlpha, PolZeta and PolDelta PolBs share a common ancestor with the archaeal B6 group. To test this hypothesis, we analyzed the domain organizations of the corresponding polymerases and investigated the provenance and evolution of the essential regulatory B-subunits of eukaryotic PolB holoenzymes.

### Two groups of archaeal PolBs have putative Zn-binding motifs at their C-termini

**Figure 6.**
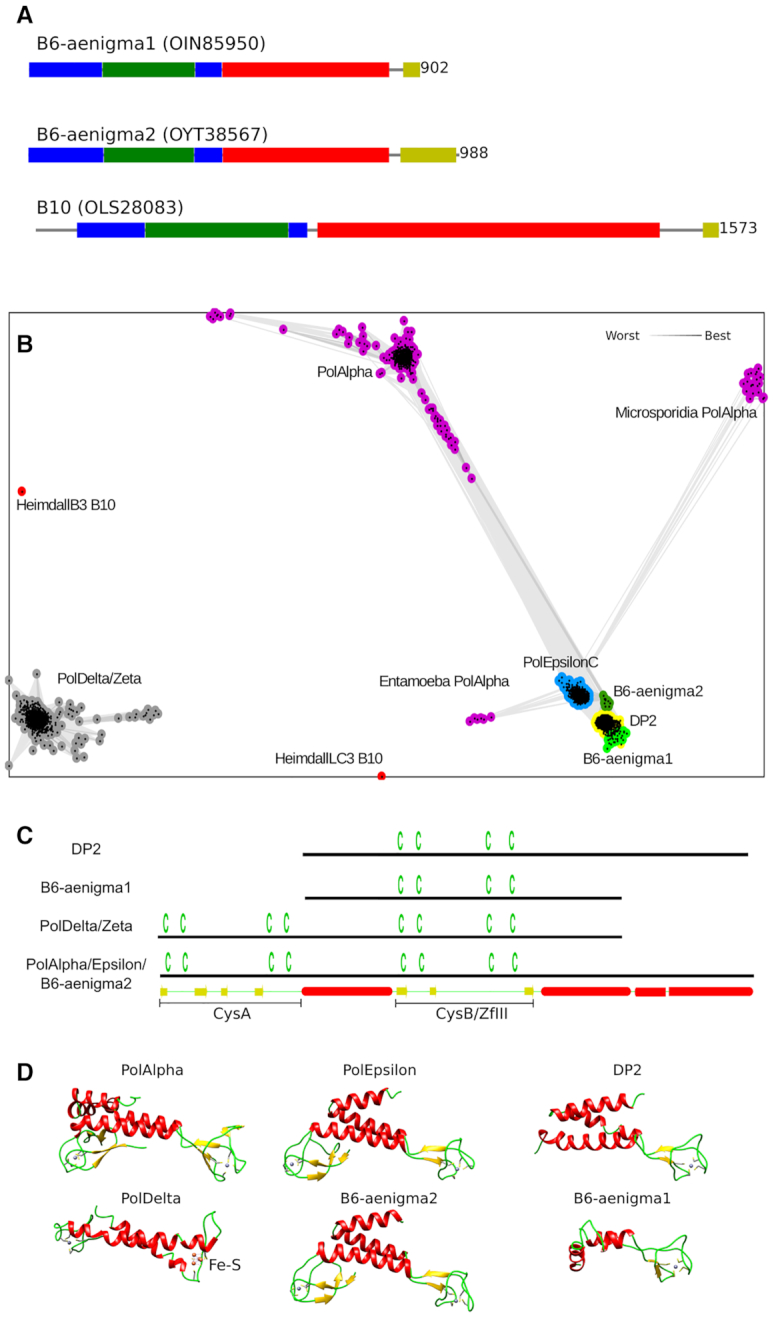
C-terminal domains (CTDs) of archaeal PolBs. (**A**) Archaeal groups of PolBs having CTD. Protein domains are shown in the same colors as in Figure 1. (**B**) Clustering of DNA polymerase CTDs. Lines connect sequences with *P*-value ≤ 1e−06. (**C**) A schematic representation of CTDs. Positions of cysteines are shown above the lines. Secondary structure of PolAlpha CTD is shown at the bottom. (**D**) CTD structures of human PolAlpha (PDB: 4y97), PolEpsilon (PDB: 5vbn), PolDelta (PDB: 6tny), *Pyrococcus abyssi* DP2 (PDB: 6t8h) and CTD homology models of B6 members, B6-aenigma1 (Acc: OIN85950) and B6-aenigma2 (Acc: RLJ05308). Structures are colored by secondary structure type (α helices, red; β sheets, yellow; coils, green). Zinc atoms are shown as gray spheres. Fe−S cluster in PolDelta CTD is labeled.

The main replicative DNA polymerase of Archaea (except for members of the phylums Crenarchaeota and Marsarchaeota) is PolD, composed of a large subunit (DP2) responsible for DNA polymerization and a smaller subunit (DP1) endowed with the proofreading activity (60). Notably, DP2 is evolutionarily unrelated to DNA polymerases of bacteria and eukaryotes, and is based on the double-psi β-barrel catalytic core found in the large subunits of the universal RNA polymerase responsible for transcription in all three domains of life (61–63). Despite dissimilarity of the core fold, DP2 and catalytic subunits of all eukaryotic PolBs share C-terminal domain (CTD). Moreover, CTD of DP2 hosts a cystein-rich zinc-binding motif corresponding to the CysB motif in eukaryotic enzymes (14,62). It should be noted that no archaeal PolBs with similar metal-binding motifs were known thus far (24). Intriguingly, after performing a comprehensive analysis of domain architectures of PolBs we identified CTDs with cystein-rich metal-binding motifs in members of group B10 (present in two MAGs of Heimdallarchaeota) and some sequences of group B6 (coming mostly from Aenigmarchaeota and metagenomic sequences). B10 sequences have one, whereas B6 sequences have either one (B6-aenigma1) or two (B6-aenigma2) metal-binding motifs (Figure 6A). Using CLANS, we clustered these newly detected CTDs together with corresponding domains of catalytic subunits of both eukaryotic PolBs and archaeal PolDs (Figure 6B). CTDs from group B10 (Heimdallarchaeota) did not cluster with the others. In contrast, CTDs from archaeal group B6 clustered with PolEpsilon, PolAlpha and DP2, most tightly with the latter. The CTD from B6-aenigma2 group, similarly to PolEpsilon and PolAlpha, has two metal-binding motifs. Based on sequence and structure similarity with PolEpsilon, PolAlpha and DP2, both motifs are expected to bind zinc. B6-aenigma2 group consists of nine metagenomic sequences, two of which are annotated to originate from Aenigmarchaeota archaeon (RLJ05308) and *Thermofilum* sp. Ex4484_82 (OYT28452) (Supplementary Table S7). The latter is most probably a recent transfer from Aenigmarchaeota because PolBs of these archaea are found as the best BLAST hits when OYT28452 is used as a query. The CTD in B6-aenigma1 group has only one metal-binding motif, most similar to the zinc-binding motif of DP2 CTD (Figure 6 and Supplementary Figure S10). Interestingly, in most sequenced Aenigmarchaeota the catalytic PolD subunit (DP2) is absent (Figure 5A). This suggests that in Aenigmarchaea the DP2 subunit of the PolD replicase has been replaced by this new form of PolB (most likely from a mobile genetic element) (Supplementary Figure S11C).

Clustering results for newly identified CTDs in B10 and B6 may be assessed by considering how well the clustering procedure reproduces the relationship between known structures. Thus, CTDs of PolAlpha, PolEpsilon and DP2 were also linked with each other, whereas PolDelta and PolZeta formed a separate cluster. These results are in line with the observed structural similarities (Figure 6). CTDs of PolAlpha, PolEpsilon and DP2 have a common three-helix bundle, while CTDs of PolDelta and PolZeta have only a pair of helices. In addition, the second metal-binding motif (CysB) in both PolDelta and PolZeta binds Fe−S cluster instead of a zinc ion as in PolAlpha/Epsilon (19,21). Most parsimonious explanation of these observations is that PolZeta/Delta PolBs have a highly divergent version of PolAlpha/Epsilon CTD. Consistent with this view, the link between PolZeta/Delta and PolAlpha/Epsilon CTDs can be established, but only via sensitive sequence profile-based searches (Supplementary Figure S12). Alternatively, one or more structural motifs (e.g. CysB) in PolDelta/Zeta may have been replaced after differentiation from PolAlpha/Epsilon.

Interestingly, CLANS clustering also revealed that CTDs of PolAlpha in Entamoeba and Microsporidia are more similar to PolEpsilon CTD than they are to PolAlpha CTD of other organisms (Figure 6B). To investigate these relationships further, we analyzed the presence/absence of PolBs in eukaryotic genomes. Unexpectedly, it turned out that all Entamoebas and some Microsporidia lack either entire PolEpsilon or its inactivated C-terminal half (Supplementary Table S8).

To investigate possible origins of the CTDs of DNA polymerases, we performed HHpred searches using B10 and B6-aenigma2 CTDs as well as the three Zn-binding motifs of DP2, DP2zfI-III (62), as queries. In all cases, Zn-binding domains of archaeal DNA-directed RNA polymerase subunit P (RpoP) and its eukaryotic homolog (RNA polymerase subunit Rpb12) were retrieved among the top-scoring hits (Supplementary Figures S13 and S14). In addition, many other Zn-finger containing proteins produced high scores, including transcription factor IIE subunit alpha, lysine biosynthetic amino acid carrier protein LysW, *E. coli* protein YfgJ and nucleolar RNA-binding protein Nop10p. To better understand the relationship between these top hits we additionally performed their all-to-all structural comparison and grouped them according to structural similarity (Supplementary Figure S11, Supplementary Table S9). The major observation based on the structural comparison is that all three DP2 zinc-binding motifs display close structural similarity, whereas PolAlpha/Epsilon CysA and CysB motifs are more similar to distinct sets of proteins than to each other. Taken together, sequence and structure comparison results suggest a scenario where the progenitor of DP2 has captured a Zn-binding motif similar to those of RpoP/Nop10p/TFIIEα, followed by its triplication. B6-aenigma1 Zn-binding motif and the second Zn-binding motif in B6-aenigma2 corresponding to CysB in PolEpsilonC/Alpha appear to have been acquired directly from DP2zfIII, likely through recombination. By contrast, the first Zn-binding motif (CysA) present in CTD of B6-aenigma2 and PolEpsilonC/Alpha has presumably originated from RpoP/LysW-like proteins in archaea and the resulting PolB was subsequently acquired by the ancestors of eukaryotes (Supplementary Figure S11).

### Phylogenetic analysis of DNA polymerase second subunits

**Figure 7.**
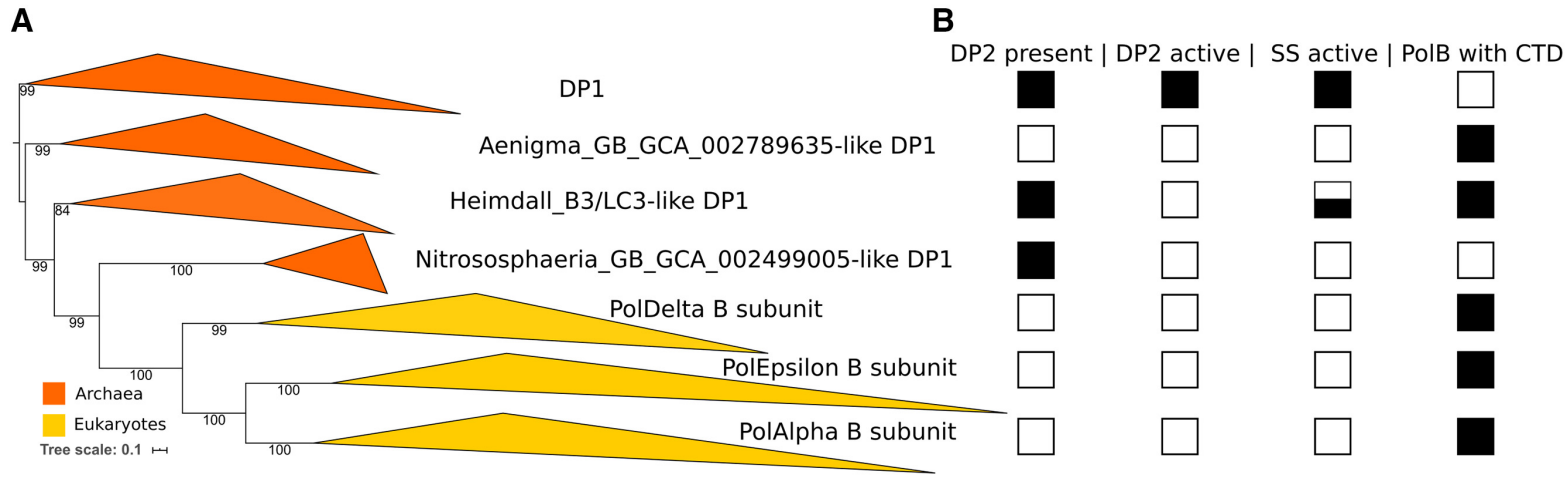
Phylogeny and properties of second subunits (SS) of DNA polymerases. (**A**) Phylogenetic tree. (**B**) Properties of catalytic subunits (DP2 or PolB) and SS that interact with them. Filled black squares indicate that all members of the group has a certain property; empty squares – none of the members; half-filled square indicates that some members (e.g. Heimdallarchaea B3) of Heimdall_B3/LC3-like group have active SS.

Evolutionarily related CTDs of catalytic subunits of eukaryotic PolBs and archaeal PolD mediate binding to the corresponding second subunits, B-subunit and DP1 (11,23,62). As in the case of CTDs, B-subunits and DP1 are homologous. Only DP1 has a phosphoesterase domain with 3′−5′ proofreading exonuclease activity, whereas in eukaryotes, this domain is inactivated (2). To better understand the evolution of archaeal and eukaryotic replicative polymerases, we performed phylogenetic analysis of their DP1/B-subunits. To collect homologs, we ran an iterative sequence search using B-subunit (p50) of human PolDelta as a query, clustered collected sequences and performed the phylogenetic analysis. Three major groups of PolDelta B-subunit homologs could be defined, namely, B-subunits of polymerases Epsilon and Alpha, and DP1 of PolD. Similarly to what was observed for the CTDs, B-subunits of PolAlpha and PolEpsilon belong to the same clade (Figure 7A). There are three groups of archaeal sequences related to the DP1, namely, Nitrososphaera_GB_GCA_002499005-like, Heimdall_B3/LC3-like and Aenigma_GB_GCA_002789635-like. Those groups differ from the major DP1 clade in that they have inactive DP1, lack active DP2, or co-occur with PolBs possessing zinc-binding motifs at their C-termini (Figure 7, Supplementary Figures S15 and S16). Presumably, inactivation of DP1 subunits in Aenigma and Heimdall_B3/LC3 could be afforded due to the acquisition of CTD-containing PolBs possessing their own proofreading exonuclease domains (B6 and B10 groups, respectively). Heimdallarchaea B3/LC3 might have acquired B10 relatively recently, because some Heimdallarchaea (e.g. AB125) do encode active DP2 and DP1 (Supplementary Figures S16 and S15, respectively). In addition, DP1 of Heimdallarchaea B3 might still be active, because it contains all five active site motifs (Supplementary Figure S15). Nitrososphaeria_GB_GCA_002499005-like group encodes an inactivated DP1, but lacks the active DP2 or PolB with CTD (Supplementary Figure S16 and Figure 7). Currently there are only two MAGs from this group (Supplementary Table S6, UBA160 and UBA164) in the GTDB and one related MAG from Thaumarchaeota (accession of DP1 − NAY82623) in the NR database. Thus, it might be premature to draw any conclusion about the evolution of this group. Notably, however, thermophilic thaumarchaea of the genus *Nitrosocaldus* lack the DP2 (64,65).

Groups Nitrososphaera_GB_GCA_002499005-like and Heimdall_B3/LC3-like are positioned at the root of the eukaryotic B-subunits in the tree, likely due to their high divergence. For example, the highest scoring homolog of the DP1 subunit of Heimdallarchaeota LC3 (OLS27757) has an *E*-value = 1e−13. B-subunits of eukaryotic PolBs might have originated from the ancestors of the Aenigma_GB_GCA_002789635-like group. However, one cannot exclude the possibility that eukaryotic PolB catalytic subunits from the Delta-like clade and the B6 group originated from related mobile genetic elements which independently introduced the ancestors of these polymerases and corresponding B-subunits in proto-eukaryotes and in some archaea.

## DISCUSSION

With six families already described (62,66,67), the diversity of DNA polymerases contrasts the uniqueness of ribosomes or the existence of only two families of non-homologous RNA polymerases, raising questions about the origin and evolution of the DNA replication machinery (66,68,69). The evolutionary history of family B DNA polymerases appears extremely convoluted. In particular, mixing of viral and cellular sequences in phylogenetic analyses of PolBs presented herein and those reported previously (3,24,66) suggests that many transfers of these enzymes have taken place between cells and viruses (in either direction), possibly explaining the absence of congruence between the tree of these DNA polymerases and the universal tree of life (66).

Here, we have focused on one of the three mechanistically defined subgroups of PolBs, the one that depends on RNA or DNA primer, to update our knowledge on the diversity and evolution of these enzymes in the age of genomics and metagenomics. We identified five major clusters of these enzymes and focused on the largest one that includes PolBs encoded by all cellular domains and viruses. Our results not only confirm the remarkable diversity of the PolBs and their wide distribution among both cells and viruses, but also greatly extend this diversity with the discovery of several new subfamilies. We discovered a new group of PolBs widespread in Bacteria and defined six new archaeal PolB groups, two of which contain a C-terminal domain with metal-binding site(s), a feature previously thought to be specific to eukaryotic DNA polymerases. The diversity of PolBs in Archaea (10 groups in total) is especially striking, although two of these groups (B8, B9) are only present in MAGs and hence their archaeal origin remains to be confirmed. Overall, the diversity of DNA polymerases in Archaea correlates well with the plasticity of the archaeal replication apparatus deduced from phylogenomic analysis (26).

Mapping the distribution of different PolB subfamilies analyzed in this work on the tree of life provides insights into the replicative machineries of the three last common ancestors of the modern domains of life. The unequivocal archaeal origin of the bacterial G2 group and the limited distribution of the G1 and G3 groups among bacterial phyla (Supplementary Figure S7) suggest that the Last Bacterial Common Ancestor (LBCA) did not encode PolB and that all three groups, G1−G3, were introduced independently in this domain, probably from viruses. Consistent with this idea, in phylogenetic analyses, G1 (including *E. coli* PolII) and G3 cluster with PolBs encoded by T4-like and crAss-like phages, respectively (Figure 3). Thus, the LBCA most likely employed family C DNA polymerase as the replicase, because this enzyme is present in all lineages of contemporary Bacteria (70).

In the case of Archaea, the distribution of PolB in genomes and MAGs suggests that the Last Archaeal Common Ancestor (LACA), besides the replicative PolD polymerase, possessed two *polB* copies, corresponding to the ancestors of groups B3 and B1/B2, respectively. The other archaeal PolBs, B4−B10, have a very limited distribution (Figure 5A) and were most likely introduced within particular archaeal branches from extinct cellular lineages or from mobile genetic elements (plasmids or viruses). Indeed, PolBs and divergent versions of other replicative proteins, such as primases, are encoded by several families of archaeal viruses and other types of mobile genetic elements integrated in archaeal genomes (5,24,71). Furthermore, introduction of new DNA replication proteins from mobile genetic elements in Archaea, with occasional non-orthologous replacement of the ancestral cellular enzyme, has been reported in the case of replication initiation protein Cdc6 and the replicative helicase MCM (26,72). A similar replacement of the cellular PolB with a viral homolog is especially likely in the case of the group B5, which among cellular organisms is restricted to halophilic archaea, but is conserved in T4-like viruses, including haloarchaeal head-tailed viruses and Magroviruses (32).

**Figure 8.**
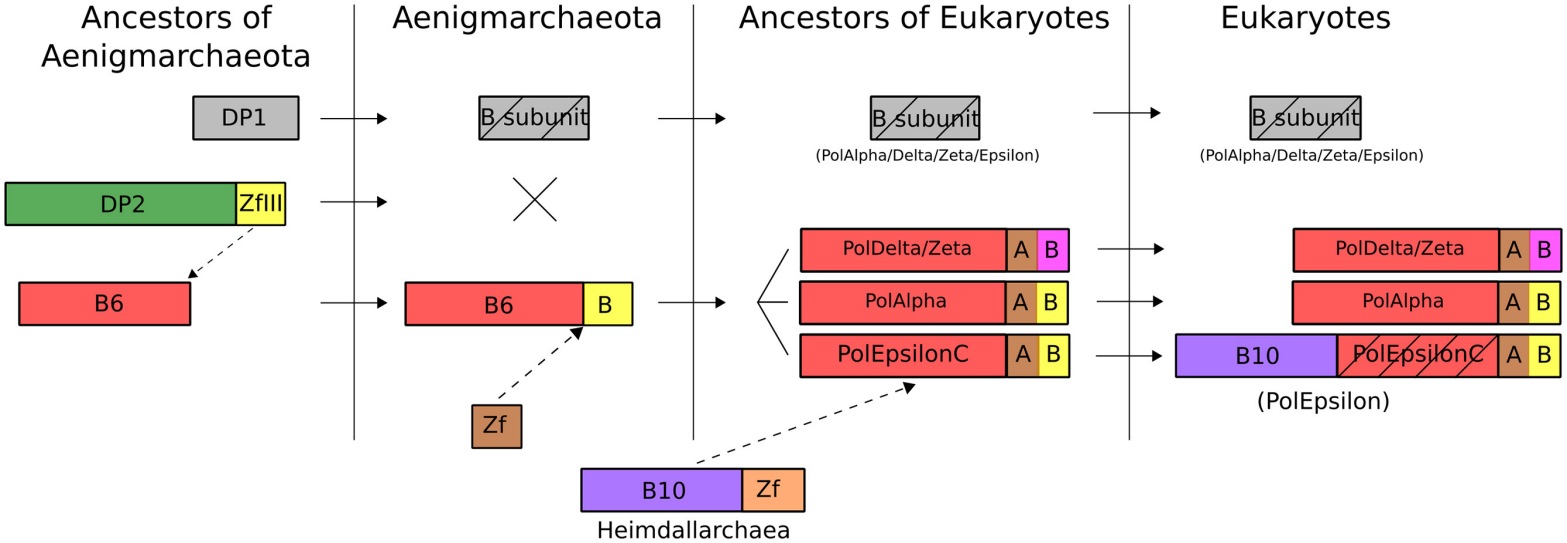
Proposed origin and evolution of catalytic and B-subunits of eukaryotic PolBs. DP2 and DP1 are correspondingly catalytic and second subunits of PolD. B6 and B10 are archaeal PolBs; B-subunit is a second subunit of DNA polymerase; Zf, zinc finger. ‘A’ and ‘B’ represent metal-binding motifs corresponding to CysA and CysB in CTD of eukaryotic PolBs, different color of PolDelta/Zeta CysB denotes Fe−S binding instead of zinc ion. The broken arrow indicates acquisition of a domain; two crossed lines, deletion of a gene; straight arrow, inheritance of a gene; rectangle with diagonal lines, inactivated domain.

In the case of eukaryotes, the wide distribution of PolAlpha, PolDelta, PolZeta and PolEpsilon subfamilies across eukaryotic supergroups (Supplementary Table S8) suggests that the Last Eukaryotic Common Ancestor (LECA) already possessed the ancestors of all four eukaryotic PolBs (Figure 8). This is in agreement with the consensus view that LECA was already a very complex cell displaying many of the features typical of modern eukaryotes, including mitochondria, elaborate endomembrane system, nucleus, etc (73). However, we highlight the secondary loss of PolEpsilon or its inactivated C-terminal half in all Entamoebas and some Microsporidia. Our phylogenetic analysis suggests that Alpha, Delta and Zeta PolBs evolved through duplication of the gene encoding the ancestral form of these enzymes, although secondary recruitment of some of them from viruses of the EukVir2–3 group cannot be excluded, as recently suggested for the eukaryotic RNA polymerases II and III (74). PolEpsilon, which is a fusion of two separate PolB modules (corresponding to PolEpsilonN and PolEpsilonC, respectively), apparently also evolved in the stem branch leading to LECA. Although PolEpsilonC is too divergent to be included in phylogenetic analysis, sensitive sequence searches and structure comparisons suggest that it is related to the aforementioned eukaryotic polymerases and has likely also evolved by gene duplication. The provenance of this pre-eukaryotic PolB gene remains elusive, however. By contrast, the N-terminal half of PolEpsilon, PolEpsilonN, has been acquired horizontally, likely from an archaeon related to Heimdallarchaea subgroup LC3/B3 (Figure 8), which forms a sister group to eukaryotic PolEpsilonN (Figure 3).

Collectively, our results further clarify the origin of eukaryotic DNA polymerases and their relationships with archaeal PolBs. In particular, eukaryotic PolBs do not emerge from within the major clades (B1–3) of archaeal PolBs. This relationship seemingly eliminates a simple scenario under which eukaryotic PolBs are direct descendants of their archaeal counterparts. Nevertheless, the eukaryotic PolAlpha, PolDelta, and PolZeta form a clade with the minor groups of archaeal PolB present in Aenigmarchaeota (B6) and group II methanogens (B4) (Figure 3). Remarkably, B6 polymerases and the eukaryotic enzymes share the C-terminal domain, not found in any other group of archaeal PolBs, validating the results of phylogenetic analysis. The cellular context of the acquisition of the B4/B6-like PolBs by the ancestor of eukaryotes remains unclear, because none of the currently postulated models for the origins of eukaryotes involves aenigmarchaea (75,76). The restricted distribution of the B4 and B6 PolB groups in Archaea suggests that they are of viral provenance.

It has been suggested that DNA polymerases, similarly to other enzymes involved in DNA transactions, originated and evolved in a greater viral world that predated the last universal cellular ancestor (LUCA) of the three modern domains and that only some of them were later on transferred to the ancestors of modern cellular domains (77,78). To explain the presence of homologs of archaeal/eukaryotic DNA replication proteins encoded by mobile genetic elements in the bacterial domain, it has been proposed that LUCA had a PolB-based DNA replication machinery (3). More recently, it has been proposed that LUCA replicated its genome by the heterodimeric PolD-like polymerase, which was subsequently replaced by PolC in bacteria (1). In the lineage leading to eukaryotes, only the DNA polymerization domain of the large PolD subunit, DP2, was substituted with PolB, while the C-terminal domain of DP2 as well as the inactivated DP1 subunit were retained (1). Our results are consistent with this scenario and extend it further by showing that all these changes could have occurred already in archaea, specifically, in the B6 group present in aenigmarchaea. Furthermore, we find that DP1 which was inherited by eukaryotes might have been inactivated already in archaea, as is the case in the above mentioned lineage of aenigmarchaea (Figure 8). Further mining of viral and archaeal genomes and metagenomes for new PolBs combined with detailed phylogenetic analysis of the different PolB families should provide even deeper understanding on the evolution of this profoundly important group of DNA polymerases.

## Supplementary Material

gkaa760_Supplemental_Files
