# Supplementary material for: Diversity and evolution of B-family DNA polymerases
Source: Nucleic Acids Res. 2020 Sep 25;48(18):10142–56. doi: 10.1093/nar/gkaa760 (PMC7544198; doi:10.1093/nar/gkaa760)
Supplement: gkaa760_Supplemental_Files [file gkaa760_supplemental_files.zip › Supplementary_Figures.docx]

**Supplementary Figures**

**Diversity and evolution of B-family DNA polymerases**

Darius Kazlauskas^1,^*, Mart Krupovic^2^, Julien Guglielmini^3^, Patrick Forterre^2^, Česlovas Venclovas^1,^*

^1^ Institute of Biotechnology, Life Sciences Center, Vilnius University, Saulėtekio av. 7, Vilnius, 10257, Lithuania

^2^ Archaeal Virology Unit, Department of Microbiology, Institut Pasteur, 25 rue du Docteur Roux, Paris 75015, France

^3^ Hub de Bioinformatique et Biostatistique – Département Biologie Computationnelle, Institut Pasteur, USR 3756 CNRS, Paris, France

* To whom correspondence should be addressed. Email: darius.kazlauskas@bti.vu.lt, ceslovas.venclovas@bti.vu.lt


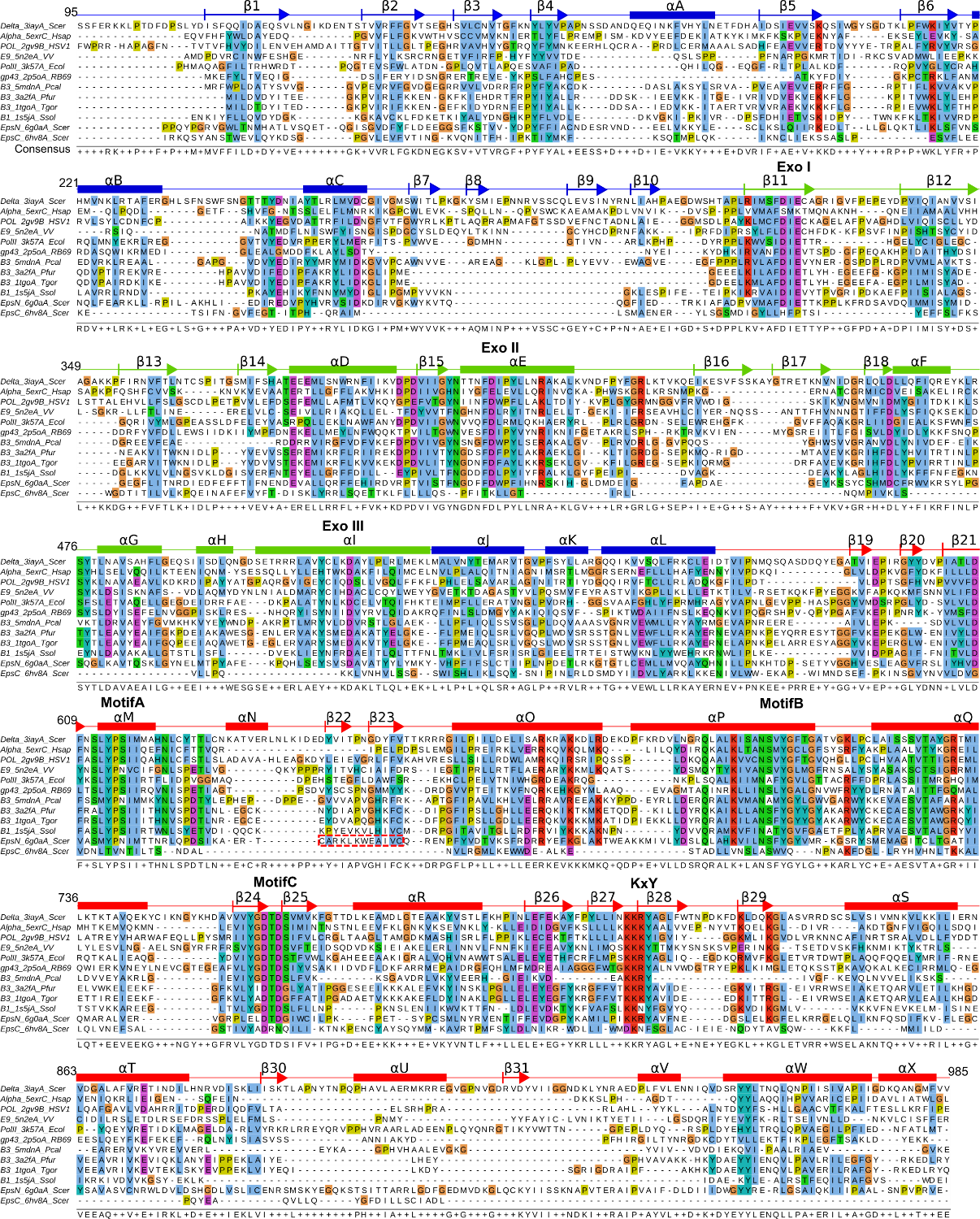


**Figure S1**. Dali structure-based sequence alignment of PolBs. Gappy regions were removed from PolDelta by Dali server. Mutated active site residues changed to wild type. PolEpsilonN has cysteine residues in the same loop as in archaeal B1 and B3 polymerases. It was suggested that in archaeal PolBs these residues are important for thermostability ([1](#_ENREF_1)). In yeast PolEpsilon it was shown that CysX motif (PolEpsilonN (NP_014137), positions C665, C668, C677 and C763 (only C677 and C763 are shown in the alignment)) bind Fe-S cluster and their mutants are inviable ([2](#_ENREF_2)). CysX region is enclosed in a red rectangle. Secondary structure of PolDelta is shown above the alignment. N-terminal, Exonuclease and DNA polymerase domains are colored blue, green and red, respectively. The active site motifs are listed above the alignment. Positions of PolDelta are marked above the alignment (left side).

**Figure S2.** Dendrogram representation of PolBs structural similarities generated by Dali server ([3](#_ENREF_3)). PDB ID and chain are shown at the tips of the branches. Most of the structures of archaeal PolBs belong to the B3 group and correspond to PolBs from both Euryarchaeota (genera Pyrococcus and Thermococcus) and Crenarchaeota (Desulfurococcus and Pyrobaculum) phyla.

**(A)**

No: Chain Z rmsd lali nres %id PDB Description

[1](http://ekhidna2.biocenter.helsinki.fi/barcosel/tmp/6hv8A/6hv8A-50.html#alignment-1): 6hv8-A 44.7 0.0 774 774 100 [PDB](http://ekhidna2.biocenter.helsinki.fi/cgi-bin/daliviewer/readpdb.cgi?html=1&jobid=pdb&pdbid=6hv8&u=1.000000,-0.000000,-0.000000,0.000000,1.000000,0.000000,0.000000,0.000000,1.000000&t=0,-0,-0) MOLECULE: DNA POLYMERASE EPSILON SUBUNIT B;

[2](http://ekhidna2.biocenter.helsinki.fi/barcosel/tmp/6hv8A/6hv8A-50.html#alignment-2): 5vbn-B 12.9 1.5 116 125 29 [PDB](http://ekhidna2.biocenter.helsinki.fi/cgi-bin/daliviewer/readpdb.cgi?html=1&jobid=pdb&pdbid=5vbn&u=0.732152,-0.053066,-0.679071,-0.616899,-0.474326,-0.628053,-0.288773,0.878749,-0.380015&t=90,239,24) MOLECULE: DNA POLYMERASE EPSILON SUBUNIT 2;

[3](http://ekhidna2.biocenter.helsinki.fi/barcosel/tmp/6hv8A/6hv8A-50.html#alignment-3): 4fxd-A 11.3 6.4 459 848 10 [PDB](http://ekhidna2.biocenter.helsinki.fi/cgi-bin/daliviewer/readpdb.cgi?html=1&jobid=pdb&pdbid=4fxd&u=-0.257451,-0.251177,0.933075,0.568763,0.741242,0.356468,-0.781171,0.622471,-0.047973&t=115,109,137) MOLECULE: DNA POLYMERASE ALPHA CATALYTIC SUBUNIT A;

[4](http://ekhidna2.biocenter.helsinki.fi/barcosel/tmp/6hv8A/6hv8A-50.html#alignment-4): 5exr-C 11.1 5.5 506 1057 10 [PDB](http://ekhidna2.biocenter.helsinki.fi/cgi-bin/daliviewer/readpdb.cgi?html=1&jobid=pdb&pdbid=5exr&u=0.445322,-0.620593,-0.645409,-0.810898,0.026102,-0.584606,0.379648,0.783699,-0.491613&t=146,203,18) MOLECULE: DNA PRIMASE SMALL SUBUNIT;

[5](http://ekhidna2.biocenter.helsinki.fi/barcosel/tmp/6hv8A/6hv8A-50.html#alignment-5): 1tgo-A 9.8 6.5 306 773 7 [PDB](http://ekhidna2.biocenter.helsinki.fi/cgi-bin/daliviewer/readpdb.cgi?html=1&jobid=pdb&pdbid=1tgo&u=0.080418,0.929748,0.359308,0.858494,-0.247773,0.448996,0.506479,0.272356,-0.818108&t=83,98,126) MOLECULE: PROTEIN (THERMOSTABLE B DNA POLYMERASE);

[6](http://ekhidna2.biocenter.helsinki.fi/barcosel/tmp/6hv8A/6hv8A-50.html#alignment-6): 2p5g-B 9.7 5.5 449 756 9 [PDB](http://ekhidna2.biocenter.helsinki.fi/cgi-bin/daliviewer/readpdb.cgi?html=1&jobid=pdb&pdbid=2p5g&u=0.573012,-0.138628,0.807738,-0.741388,-0.507735,0.438803,0.349286,-0.850287,-0.393715&t=92,148,240) MOLECULE: TEMPLATE DNA;

[7](http://ekhidna2.biocenter.helsinki.fi/barcosel/tmp/6hv8A/6hv8A-50.html#alignment-7): 3iay-A 9.6 6.4 483 885 7 [PDB](http://ekhidna2.biocenter.helsinki.fi/cgi-bin/daliviewer/readpdb.cgi?html=1&jobid=pdb&pdbid=3iay&u=0.613417,-0.461167,0.641128,0.758611,0.569807,-0.315957,-0.219611,0.680180,0.699375&t=85,109,95) MOLECULE: 5'D(*AP*TP*CP*CP*TP*CP*CP*CP*CP*TP*AP*(DOC))3';

[8](http://ekhidna2.biocenter.helsinki.fi/barcosel/tmp/6hv8A/6hv8A-50.html#alignment-8): 1s5j-A 9.5 5.8 431 727 9 [PDB](http://ekhidna2.biocenter.helsinki.fi/cgi-bin/daliviewer/readpdb.cgi?html=1&jobid=pdb&pdbid=1s5j&u=-0.410630,-0.911648,-0.016770,0.480316,-0.200640,-0.853838,0.775035,-0.358667,0.520268&t=131,103,50) MOLECULE: DNA POLYMERASE I;

[9](http://ekhidna2.biocenter.helsinki.fi/barcosel/tmp/6hv8A/6hv8A-50.html#alignment-9): 5mdn-A 9.3 6.5 396 761 10 [PDB](http://ekhidna2.biocenter.helsinki.fi/cgi-bin/daliviewer/readpdb.cgi?html=1&jobid=pdb&pdbid=5mdn&u=0.827655,0.404823,-0.388724,-0.451870,0.891445,-0.033739,0.332868,0.203577,0.920736&t=22,154,133) MOLECULE: DNA POLYMERASE;

**(B)**

No: Chain Z rmsd lali nres %id PDB Description

[1](http://ekhidna2.biocenter.helsinki.fi/barcosel/tmp/838b2d3b222ec8564d34ad92b03e866ec77b2ccca34ed0b43f9ce0a8/s001A-50.html#alignment-1): 6hv8-A 40.7 0.0 658 774 100 [PDB](http://ekhidna2.biocenter.helsinki.fi/cgi-bin/daliviewer/readpdb.cgi?html=1&jobid=pdb&pdbid=6hv8&u=1.000000,-0.000040,-0.000073,0.000040,1.000000,-0.000012,0.000073,0.000012,1.000000&t=0,-0,-0) MOLECULE: DNA POLYMERASE EPSILON SUBUNIT B;

[2](http://ekhidna2.biocenter.helsinki.fi/barcosel/tmp/838b2d3b222ec8564d34ad92b03e866ec77b2ccca34ed0b43f9ce0a8/s001A-50.html#alignment-2): 5exr-C 11.8 5.5 382 1057 8 [PDB](http://ekhidna2.biocenter.helsinki.fi/cgi-bin/daliviewer/readpdb.cgi?html=1&jobid=pdb&pdbid=5exr&u=0.622717,-0.583359,-0.521456,-0.740076,-0.222794,-0.634548,0.253992,0.781060,-0.570467&t=119,220,35) MOLECULE: DNA PRIMASE SMALL SUBUNIT;

[3](http://ekhidna2.biocenter.helsinki.fi/barcosel/tmp/838b2d3b222ec8564d34ad92b03e866ec77b2ccca34ed0b43f9ce0a8/s001A-50.html#alignment-3): 6i8a-B 10.3 5.6 469 1094 8 [PDB](http://ekhidna2.biocenter.helsinki.fi/cgi-bin/daliviewer/readpdb.cgi?html=1&jobid=pdb&pdbid=6i8a&u=0.998892,-0.038006,-0.027742,-0.006298,-0.692266,0.721615,-0.046630,-0.720641,-0.691738&t=-68,22,330) MOLECULE: DNA POLYMERASE EPSILON CATALYTIC SUBUNIT A;

[4](http://ekhidna2.biocenter.helsinki.fi/barcosel/tmp/838b2d3b222ec8564d34ad92b03e866ec77b2ccca34ed0b43f9ce0a8/s001A-50.html#alignment-4): 4fxd-A 10.3 6.4 458 848 10 [PDB](http://ekhidna2.biocenter.helsinki.fi/cgi-bin/daliviewer/readpdb.cgi?html=1&jobid=pdb&pdbid=4fxd&u=-0.251068,-0.260337,0.932303,0.573335,0.736029,0.359928,-0.779905,0.624889,-0.035533&t=114,109,137) MOLECULE: DNA POLYMERASE ALPHA CATALYTIC SUBUNIT A;

[5](http://ekhidna2.biocenter.helsinki.fi/barcosel/tmp/838b2d3b222ec8564d34ad92b03e866ec77b2ccca34ed0b43f9ce0a8/s001A-50.html#alignment-5): 4m8o-A 9.7 5.6 456 1126 9 [PDB](http://ekhidna2.biocenter.helsinki.fi/cgi-bin/daliviewer/readpdb.cgi?html=1&jobid=pdb&pdbid=4m8o&u=-0.666530,-0.021803,-0.745160,0.543076,-0.698961,-0.465319,-0.510693,-0.714827,0.477719&t=137,157,143) MOLECULE: DNA POLYMERASE EPSILON CATALYTIC SUBUNIT A;

[6](http://ekhidna2.biocenter.helsinki.fi/barcosel/tmp/838b2d3b222ec8564d34ad92b03e866ec77b2ccca34ed0b43f9ce0a8/s001A-50.html#alignment-6): 5mdn-A 9.5 5.9 391 761 10 [PDB](http://ekhidna2.biocenter.helsinki.fi/cgi-bin/daliviewer/readpdb.cgi?html=1&jobid=pdb&pdbid=5mdn&u=0.809027,0.415296,-0.415938,-0.485309,0.871196,-0.074107,0.331588,0.261813,0.906368&t=22,154,133) MOLECULE: DNA POLYMERASE;

[7](http://ekhidna2.biocenter.helsinki.fi/barcosel/tmp/838b2d3b222ec8564d34ad92b03e866ec77b2ccca34ed0b43f9ce0a8/s001A-50.html#alignment-7): 1tgo-A 9.0 5.5 288 773 9 [PDB](http://ekhidna2.biocenter.helsinki.fi/cgi-bin/daliviewer/readpdb.cgi?html=1&jobid=pdb&pdbid=1tgo&u=0.060047,0.934628,0.350522,0.854979,-0.229390,0.465179,0.515176,0.271756,-0.812861&t=82,98,126) MOLECULE: PROTEIN (THERMOSTABLE B DNA POLYMERASE);

[8](http://ekhidna2.biocenter.helsinki.fi/barcosel/tmp/838b2d3b222ec8564d34ad92b03e866ec77b2ccca34ed0b43f9ce0a8/s001A-50.html#alignment-8): 3k57-A 9.0 4.8 292 782 8 [PDB](http://ekhidna2.biocenter.helsinki.fi/cgi-bin/daliviewer/readpdb.cgi?html=1&jobid=pdb&pdbid=3k57&u=-0.466478,0.374999,-0.801108,-0.630599,0.494120,0.598490,0.620276,0.784361,0.005978&t=118,98,105) MOLECULE: DNA POLYMERASE II;

[9](http://ekhidna2.biocenter.helsinki.fi/barcosel/tmp/838b2d3b222ec8564d34ad92b03e866ec77b2ccca34ed0b43f9ce0a8/s001A-50.html#alignment-9): 1s5j-A 8.9 6.4 454 727 10 [PDB](http://ekhidna2.biocenter.helsinki.fi/cgi-bin/daliviewer/readpdb.cgi?html=1&jobid=pdb&pdbid=1s5j&u=-0.428383,-0.903227,0.025875,0.453457,-0.239658,-0.858453,0.781578,-0.356014,0.512240&t=131,103,50) MOLECULE: DNA POLYMERASE I;

**Figure S3**. Results of a Dali server search using PolEpsilonC as a query against the PDB database filtered to 50% identity. (A) Full length PolEpsilonC was used as a query (6hv8A). (B) PolEpsilonC without the C-terminal domain as a query. Hits to PolAlpha are shown in yellow background.

Query P21951 Saccharomyces cerevisiae strain ATCC 204508 / S288c

Match_columns 827

No_of_seqs 141 out of 462

Neff 7.36882

Searched_HMMs 89171

Date Thu Sep 19 12:07:37 2019

Command hhsearch -cpu 8 -i ../results/full.a3m -d /cluster/toolkit/production/databases/hh-suite/mmcif70/pdb70 -d /cluster/toolkit/production/databases/hh-suite/COG_KOG/COG_KOG -d /cluster/toolkit/production/databases/hh-suite/pfama/pfama -d /cluster/toolkit/production/databases/hh-suite/NCBI_CD/NCBI_CD -o ../results/9014894_4.hhr -oa3m ../results/9014894_4.a3m -p 20 -Z 250 -loc -z 1 -b 1 -B 250 -ssm 2 -sc 1 -seq 1 -dbstrlen 10000 -norealign -maxres 32000 -contxt /cluster/toolkit/production/bioprogs/tools/hh-suite-build/data/context_data.crf

No Hit Prob E-value P-value Score SS Cols Query HMM Template HMM

1 KOG1798 DNA polymerase epsilon 100.0 1E-134 2E-139 1335.7 91.5 800 1-827 1229-2036(2173)

2 6HV8_A DNA polymerase epsilon 100.0 1E-133 1E-138 1242.4 85.9 785 43-827 1-785 (914)

3 PF08490.12 ; DUF1744 ; Domain 100.0 5.5E-80 6.1E-85 695.7 42.4 376 263-655 1-397 (397)

4 4FVM_A DNA polymerase alpha ca 96.8 0.0049 5.5E-08 80.4 21.3 245 369-656 411-705 (910)

5 4QCL_A DNA polymerase alpha ca 96.8 0.0049 5.5E-08 80.5 20.3 252 369-655 415-723 (922)

6 5EXR_C DNA primase small subun 96.7 0.0098 1.1E-07 79.5 22.0 254 369-662 416-731 (1128)

7 COG0417 PolB; DNA polymerase e 96.6 0.013 1.5E-07 74.8 20.6 249 369-659 341-623 (792)

8 KOG0969 DNA polymerase delta, 96.4 0.012 1.3E-07 78.2 18.6 259 369-660 481-781 (1066)

9 PF00136.21 ; DNA_pol_B ; DNA p 96.3 0.012 1.3E-07 71.0 15.9 229 393-658 1-290 (465)

10 6Q4T_A DNA polymerase/DNA Comp 96.3 0.026 2.9E-07 72.5 19.6 249 364-655 310-597 (774)

11 3K59_A DNA polymerase II (E.C. 96.1 0.079 8.9E-07 68.3 21.8 238 369-659 340-627 (786)

12 KOG0970 DNA polymerase alpha, 96.1 0.061 6.9E-07 73.8 21.5 272 369-660 742-1041(1429)

13 5N2E_A DNA polymerase (E.C.2.7 96.1 0.024 2.7E-07 75.0 17.2 252 369-660 468-818 (1010)

14 cd05532 POLBc_alpha; DNA polym 96.0 0.0078 8.7E-08 70.9 11.1 112 547-660 113-224 (400)

15 3IAY_A DNA polymerase delta ca 95.9 0.099 1.1E-06 68.8 20.8 252 369-660 456-758 (919)

16 2GV9_A DNA polymerase (E.C.2.7 95.9 0.15 1.7E-06 68.8 22.2 254 369-656 541-903 (1193)

17 cd05533 POLBc_delta; DNA polym 95.9 0.015 1.7E-07 68.3 11.7 112 547-659 116-236 (393)

18 cd00145 POLBc; DNA polymerase 95.8 0.034 3.9E-07 63.6 13.8 110 544-656 107-217 (323)

19 5MDN_A DNA polymerase (E.C.2.7 95.7 0.081 9.1E-07 68.1 17.7 241 364-656 331-609 (783)

20 cd05536 POLBc_B3; DNA polymera 95.2 0.039 4.3E-07 64.5 9.9 106 547-659 110-217 (371)

21 KOG0968 DNA polymerase zeta, c 94.7 1.1 1.2E-05 62.6 21.2 267 369-660 879-1197(1488)

22 cd05538 POLBc_Pol_II_B; DNA po 94.6 0.14 1.5E-06 59.6 10.8 104 547-655 87-192 (347)

23 cd05537 POLBc_Pol_II; DNA poly 94.5 0.32 3.6E-06 57.1 13.4 134 521-656 75-226 (371)

24 cd05534 POLBc_zeta; DNA polyme 94.4 0.22 2.5E-06 60.0 11.9 116 544-660 169-288 (451)

25 3QEX_A DNA polymerase (E.C.2.7 92.9 11 0.00012 50.4 22.6 249 369-660 329-716 (903)

26 cd05530 POLBc_B1; DNA polymera 92.7 1.1 1.2E-05 52.8 11.8 107 544-659 118-225 (372)

27 cd05531 POLBc_B2; DNA polymera 92.6 1.7 1.9E-05 50.9 12.9 129 521-660 74-212 (352)

28 1S5J_A DNA polymerase I (E.C.2 90.2 2.3 2.6E-05 55.9 11.0 105 547-660 570-675 (847)

29 4M8O_A DNA polymerase epsilon 79.2 15 0.00017 50.8 9.4 108 547-655 826-972 (1228)

30 cd05535 POLBc_epsilon; DNA pol 71.1 99 0.0011 40.0 12.6 134 521-655 229-427 (621)

31 2PY5_A DNA polymerase (E.C.2.7 27.2 1E+03 0.012 30.3 10.6 116 525-654 359-502 (575)

32 KOG1798 DNA polymerase epsilon 25.8 4.7E+02 0.0053 39.7 8.1 108 547-655 787-933 (2173)

33 5EUH_D Putative GGDEF domain m 21.7 1.4E+03 0.016 22.0 8.4 71 568-638 52-122 (172)

34 1UG8_A Poly(A)-specific Ribonu 20.3 8.2E+02 0.0092 22.9 5.9 64 543-613 12-82 (87)

35 cd07556 Nucleotidyl_cyc_III; C 20.3 1.2E+03 0.014 20.6 8.5 59 568-626 21-79 (133)

36 1QYS_A TOP7; alpha-beta, compu 20.2 9.9E+02 0.011 22.3 5.9 45 575-626 29-73 (106)

**Figure S4**. Results of the HHpred search using PolEpsilonC (P21951:1266-2092) of *Saccharomyces cerevisiae* (strain ATCC 204508 / S288c) as a query. Hits to PolAlpha profiles are indicated with yellow background. The first three hits are the PolEpsilonC profiles (self hits).


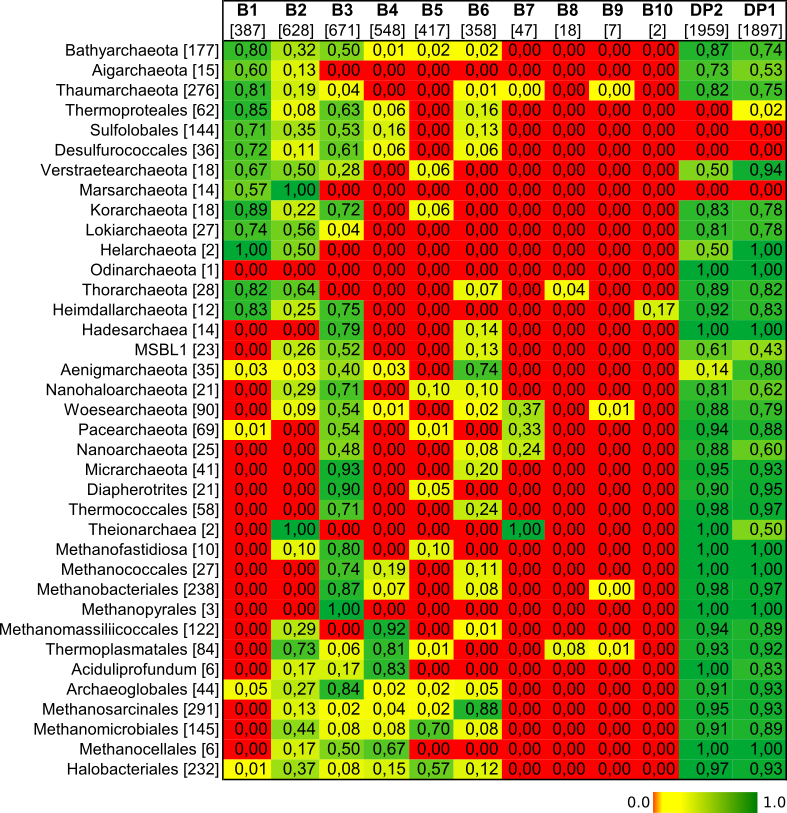


**Figure S5**. Distribution of PolBs, PolD large (DP2) and small (DP1) subunits in archaeal genomes from Genbank (Table S4). Numbers of species in a taxon and of sequences in a specific group are shown in brackets. The numbers in the cells indicate the fraction of genomes in which the corresponding protein genes were found.

**Figure S6.** Conserved motifs of PolBs. Motifs were derived using WebLogo from the alignment used to construct the phylogenetic tree shown in Figure 3. Names of motifs/regions are taken from literature ([4](#_ENREF_4),[5](#_ENREF_5)) or are named after the secondary structure elements of PolDelta (Figure S1) (1, β4; 2, αC; 3, ExoI; 4, ExoII/RegionIV; 5, αF; 6, ExoIII; 7, D(K/R) - a putative polymerase active site motif of B2/G2 polymerases; 8, MotifA/RegionII; 9, RegionVI; 10, MotifB/RegionIII; 11, MotifC/RegionI; 12, MotifKxY; 13, RegionV; 14, β31; 15, αW). Not aligned regions six residues and longer are marked with ‘-’ sign. Colors of motif numbers correspond to domains (N-terminal, blue; Exonuclease, green; Polymerase; red).

**Figure S7**. Distribution of PolBs in bacterial phyla. Only phyla having at least 20 species are shown. The number of species in a taxon and the number of members in a group are shown in brackets. The boxes are shaded by the proportion of members having PolBs in a phyla (white, [0:0.1); light grey, [0.1:0.3); dark grey, [0.3:0.6); black, [0.6:1]).


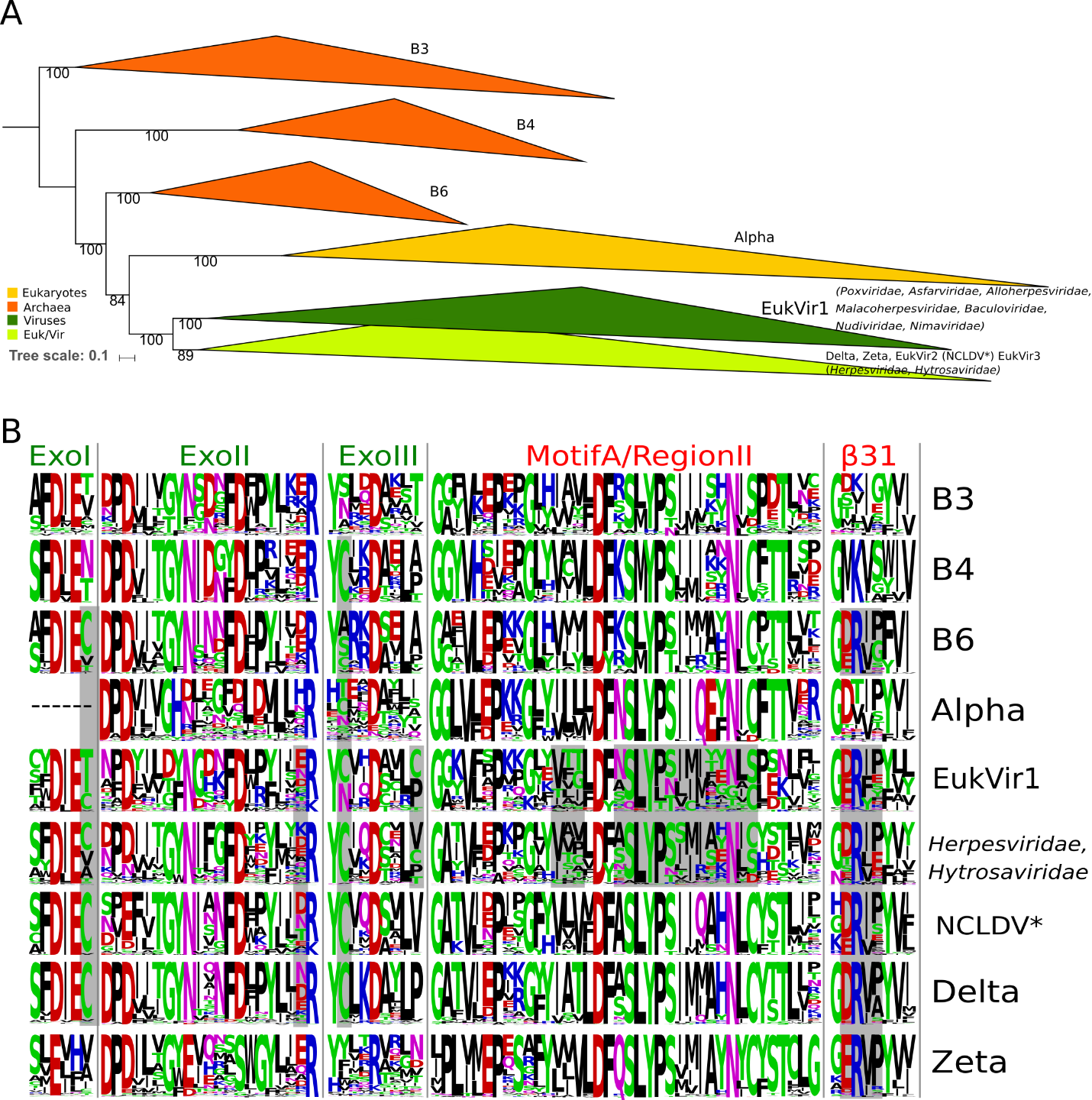


**Figure S8.** Delta-like clade and its homologs. (A) Phylogenetic tree and (B) conserved motifs. Similar positions between EukVir1 and groups from Delta-like clade are shown in gray background. Asterisk marks a group of sequences from NCLDV except *Poxviridae* and *Asfarviridae*.

**Figure S9**. Phylogenetic tree of PolBs based on MAFFT alignment with the inclusion of structural information (see Methods). Asterisk marks a group of sequences from NCLDV except *Poxviridae* and *Asfarviridae*.


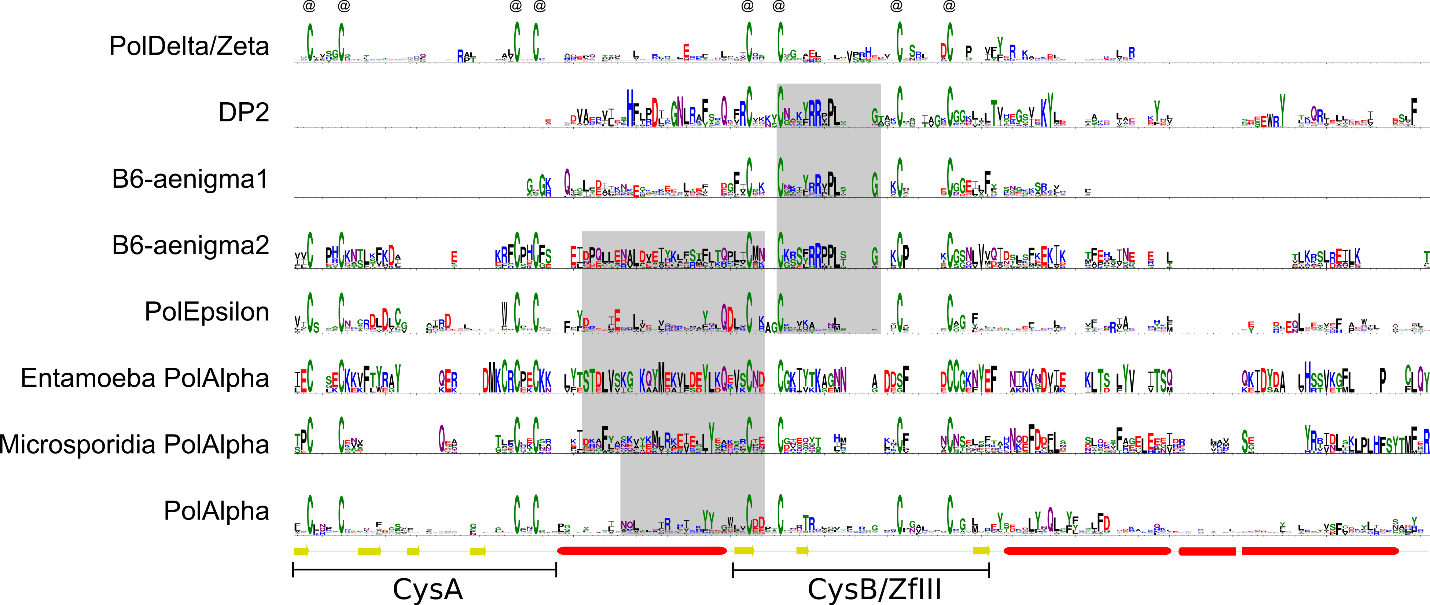


**Figure S10.** Weblogos of DNA polymerase C-terminal domains. Similar motifs are shown in gray background. Secondary structure of PolAlpha (PDB:5exr_C) is shown at the bottom; @, conserved cysteine residues.


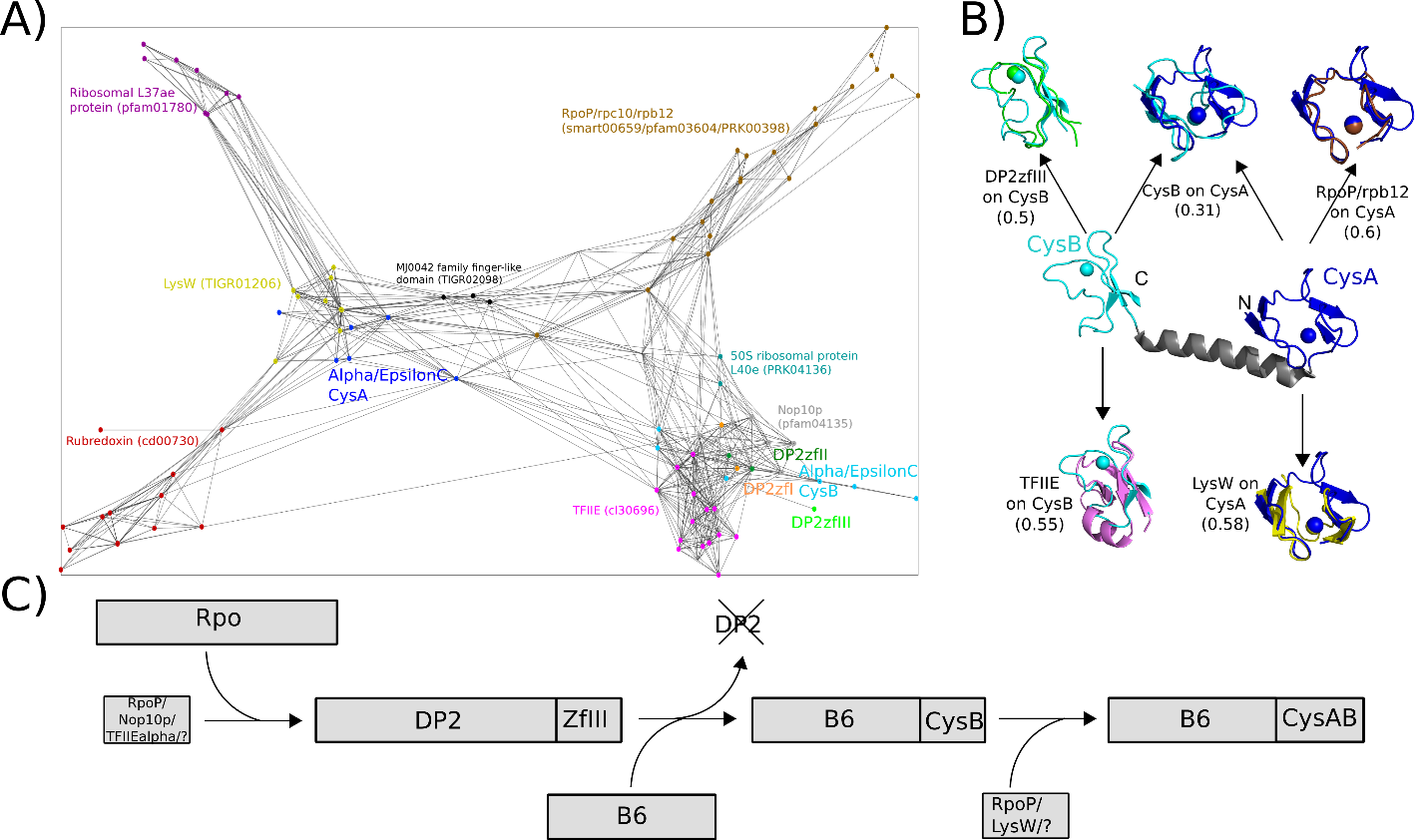


**Figure S11.** Structure similarity and possible origin of DNA polymerase C-terminal metal-binding domains (MBDs). (A) Structures of MBDs clustered by their pairwise structure similarity. Connecting lines correspond to TM-scores of 0.5 or higher. (B) Structures of MBDs (CysB and CysA) from DNA polymerase Alpha (PDB: 3floA, shown in the center) superimposed on DP2 ZfIII (6hmsB), RNA polymerase subunit (Rpb12) (5ip7L), transcription factor IIE subunit alpha (1vd4A) and lysine biosynthetic amino acid carrier protein LysW (3wwlA) using TMalign. TM-Scores are shown in parentheses. Zn atoms are shown as spheres. (C) A proposed scenario of origin and evolution of DNA polymerase MBDs.


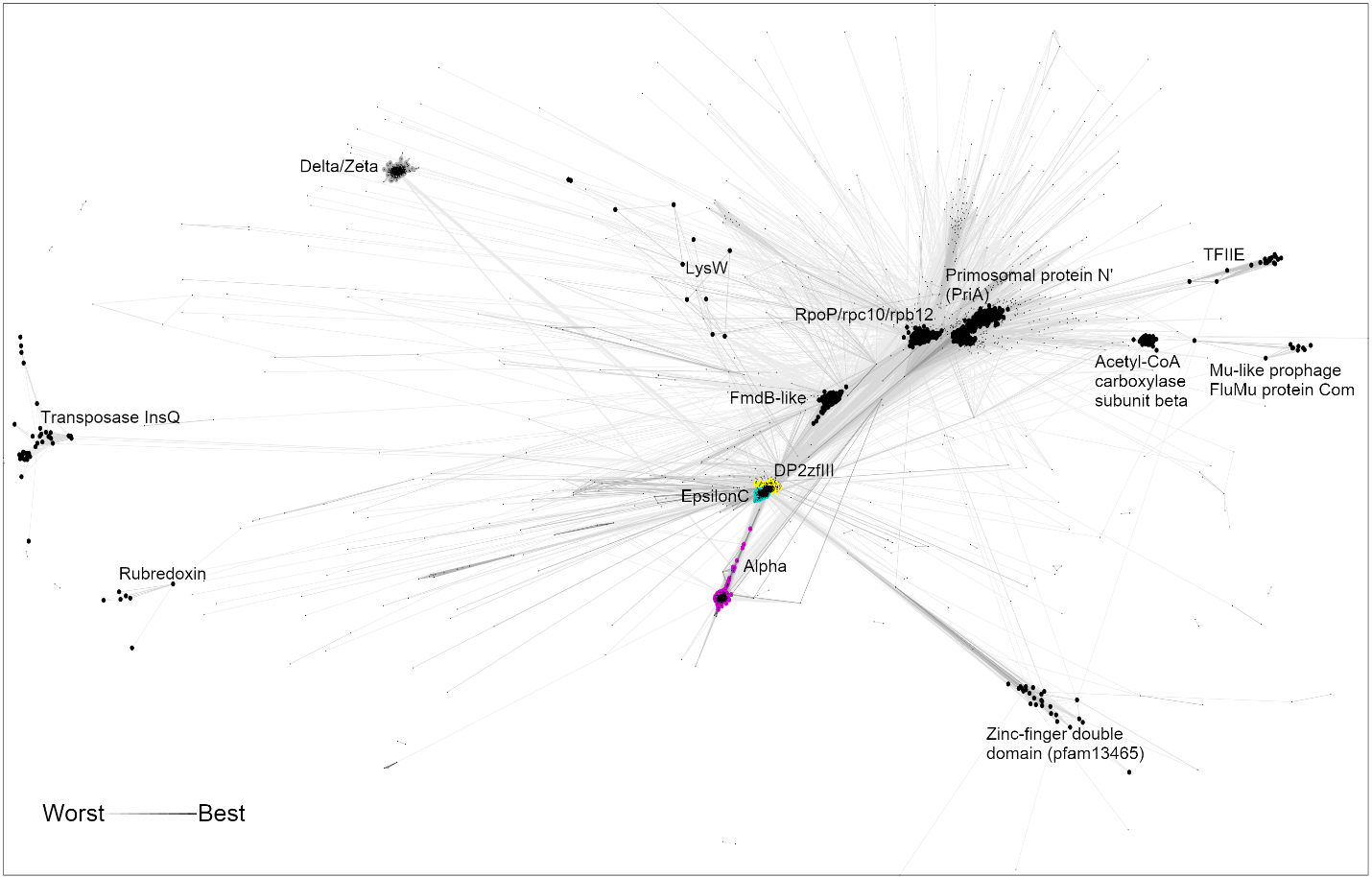


**Figure S12.** Sequence comparisons of B-family DNA polymerase CTDs, DP2 ZfIII and RpoP homologs. Homologs for DP2 ZfIII (6HMS_B:1090-1208) and RpoP (4QIW_P) were collected using three iterations of either HHblits searches against uniclust30_2018_08 database and Jackhmmer searches against uniref50 and Mgnify databases. Resulting hits were supplemented with the sequences from Figure 6B. To cluster sequences with CLANS, three PSI-BLAST iterations with the 1e-03 inclusion threshold were run against the UniRef50 database, supplemented with sequences to be compared. Clusters were identified using convex clustering algorithm and visual inspection. Lines connect sequences with P-value ≤ 1e−06.

**(A)**

No 1

>4QIW_P DNA-directed RNA polymerase (E.C.2.7.7.6), DNA-directed; Transcription, DNA-directed RNA polymerase; HET: ZN; 3.5A (Thermococcus kodakarensis); Related PDB entries: 4QIW_W

Probab=98.05 E-value=1.2e-07 Score=46.53 Aligned_cols=37 Identities=19% Similarity=0.433 Sum_probs=0.0 Template_Neff=10.700

Q ss_pred cCceeEEEECCCCCCEEEeeeC-CCCCCCCCCCCcceeeC

Q OLS28083 22 TQNYESHFTCPVCNLKFAQHQC-LPGNICPYCQKKTNCSF 60 (60)

Q Consensus 22 ~~~~~y~Y~C~~Cg~~f~~~~~-~~~~~CP~CG~~~~~~~ 60 (60)

|+. |.|+|+.||+.++.... .....||+||+.+....

T Consensus 1 Mp~--~~~~C~~C~~~~~~~~~~~~~~~Cp~Cg~~~~~~~ 38 (49)

T 4QIW_P 1 MAT--AVYRCAKCGKEVELDLATAREVRCPYCGSKILYKP 38 (49)

T ss_pred Ccc--eEEEcCCCCCEEEeCccccCceeCCCCCCcceeCC

**(B)**

No 1

>6GMH_L RPB1, DNA-directed RNA polymerase subunit; DNA, RNA Polymerase, DSIF, PAF1c; HET: TPO, SEP; 3.1A (Sus scrofa); Related PDB entries: 5IY7_L 5IY6_L 5IYD_L 5IYC_L 5OIK_L 6EXV_L 5IYB_L 5FLM_L 6GML_L 5IYA_L 5IY9_L 5IY8_L

Probab=97.78 E-value=3.2e-07 Score=52.52 Aligned_cols=36 Identities=17% Similarity=0.108 Sum_probs=0.0 Template_Neff=7.100

Q ss_pred cCCceeceEEECCCCCCccCCCccCCccCCCCCCcc

Q PWI48375 23 QVDVQADEEIVCSLCGAFISPTEVIDEGCIYCHDQP 58 (61)

Q Consensus 23 ~~~~q~~~~~KCP~CGa~l~w~g~~~~~CPYCG~~~ 58 (61)

+........|+||+||+.+.+......+||+||..+

T Consensus 8 ~~~~~~~~~y~C~~Cg~~~~~~~~~~~~Cp~CG~~~ 43 (58)

T 6GMH_L 8 QPPKQQPMIYICGECHTENEIKSRDPIRCRECGYRI 43 (58)

T ss_dssp -----CCCEEEETTTCCEEECCSSCCCCCTTTTCCE

T ss_pred CCCCCCCeEEECCCCCCEEEeccCCCccCCCCCCee

**Figure S13.** Best hits of HHpred search against PDB using CTDs from (A) Candidatus Heimdallarchaeota archaeon LC_3 (OLS28083) and (B) Candidatus Heimdallarchaeota archaeon B3_Heim (PWI48375) as queries.


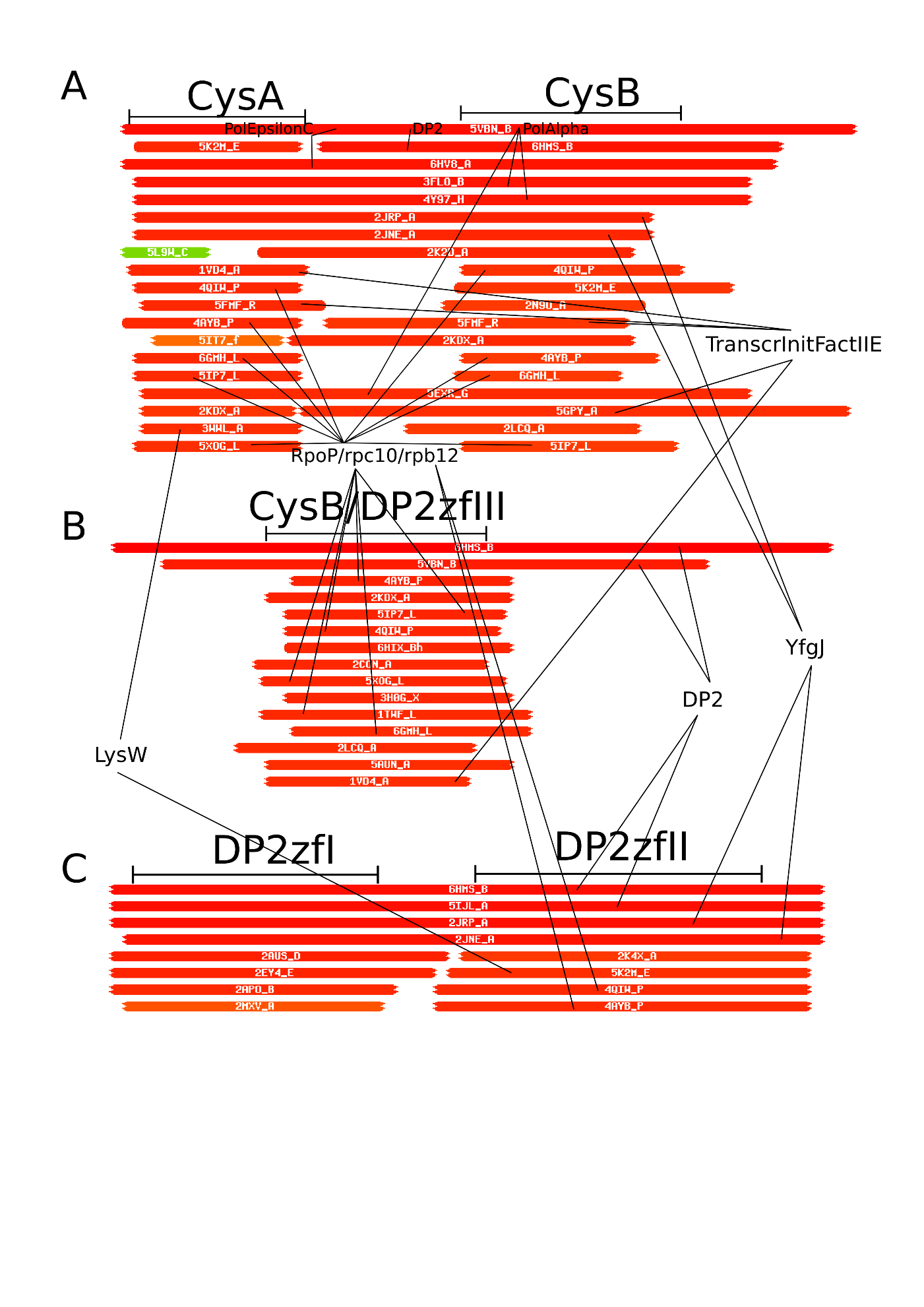


**Figure S14**. Top scoring PDB hits found by HHpred searches for (A) B6-aenigma2, (B) Paby DP2zfIII and (C) Paby DP2zfI_II.

**Figure S15.** Alignment of PolD DP1 subunits from HeimdallB3/LC3, *Heimdallarchaea AB125*, *Pyrococcus abyssi* (Paby), *Lokiarchaeum sp. GC14_75* (Loki), *Methanomassiliicoccus luminyensis* (Mlumi). Motifs involved in catalysis are marked above the alignment according to ([6](#_ENREF_6)). Sequences Nitrososphaera_GB_GCA_002499005 and Thaumarchaeota_NAY82623 belong to the Nitrososphaera_GB_GCA_002499005-like group.


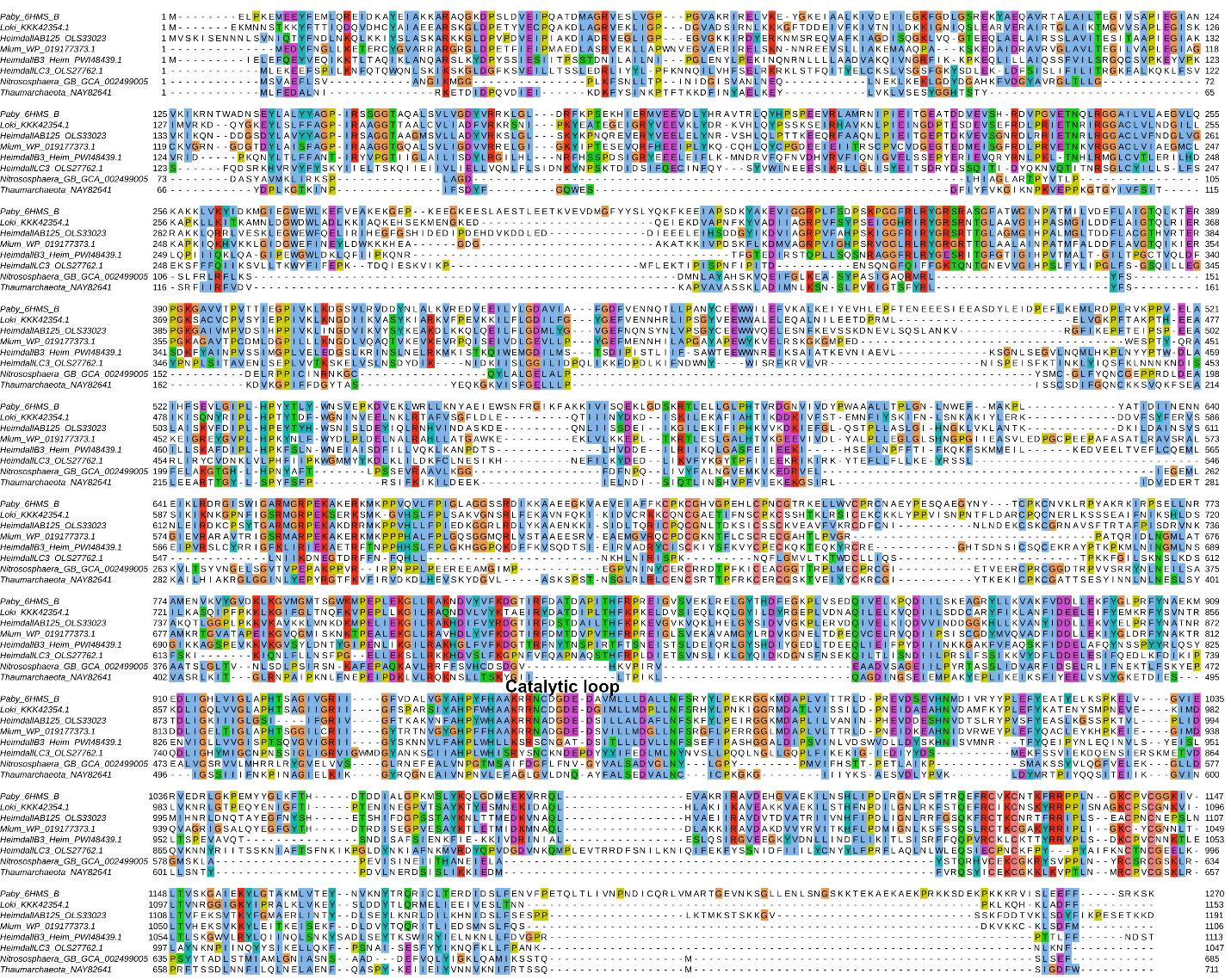


**Figure S16**. Alignment of PolD DP2 subunits from HeimdallB3/LC3, Aenigmarchaeaota, Nitrososphaeria (NAY82623 and GB_GCA_002499005) and their homologs from *Pyrococcus abyssi* (Paby), *Lokiarchaeum sp. GC14_75* (Loki), *Methanomassiliicoccus luminyensis* (Mlumi) and *Heimdallarchaea AB125*. The active site location is marked above the alignment. Sequences Nitrososphaera_GB_GCA_002499005 and Thaumarchaeota_NAY82641 belong to the Nitrososphaera_GB_GCA_002499005-like group.

**References**

1. Savino, C., Federici, L., Johnson, K.A., Vallone, B., Nastopoulos, V., Rossi, M., Pisani, F.M. and Tsernoglou, D. (2004) Insights into DNA replication: the crystal structure of DNA polymerase B1 from the archaeon Sulfolobus solfataricus. *Structure*, **12**, 2001-2008.

2. Ter Beek, J., Parkash, V., Bylund, G.O., Osterman, P., Sauer-Eriksson, A.E. and Johansson, E. (2019) Structural evidence for an essential Fe-S cluster in the catalytic core domain of DNA polymerase ϵ. *Nucleic acids research*, **47**, 5712-5722.

3. Holm, L. (2019) Benchmarking Fold Detection by DaliLite v.5. *Bioinformatics (Oxford, England)*.

4. Brautigam, C.A. and Steitz, T.A. (1998) Structural and functional insights provided by crystal structures of DNA polymerases and their substrate complexes. *Curr. Opin. Struct. Biol.*, **8**, 54-63.

5. Hopfner, K.P., Eichinger, A., Engh, R.A., Laue, F., Ankenbauer, W., Huber, R. and Angerer, B. (1999) Crystal structure of a thermostable type B DNA polymerase from Thermococcus gorgonarius. *Proc. Natl. Acad. Sci. U.S.A.*, **96**, 3600-3605.

6. Raia, P., Carroni, M., Henry, E., Pehau-Arnaudet, G., Brûlé, S., Béguin, P., Henneke, G., Lindahl, E., Delarue, M. and Sauguet, L. (2019) Structure of the DP1-DP2 PolD complex bound with DNA and its implications for the evolutionary history of DNA and RNA polymerases. *PLoS Biol.*, **17**, e3000122.
